# Neutrophil Attack Triggers Extracellular Trap-Dependent *Candida* Cell Wall Remodeling and Altered Immune Recognition

**DOI:** 10.1371/journal.ppat.1005644

**Published:** 2016-05-25

**Authors:** Alex Hopke, Nadine Nicke, Erica E. Hidu, Genny Degani, Laura Popolo, Robert T. Wheeler

**Affiliations:** 1 Molecular and Biomedical Sciences, University of Maine, Orono, Maine, United States of America; 2 Department of Biosciences, University of Milan, Milan, Italy; 3 Graduate School of Biomedical Sciences and Engineering, University of Maine, Orono, Maine, United States of America; University of Würzburg, GERMANY

## Abstract

Pathogens hide immunogenic epitopes from the host to evade immunity, persist and cause infection. The opportunistic human fungal pathogen *Candida albicans*, which can cause fatal disease in immunocompromised patient populations, offers a good example as it masks the inflammatory epitope β-glucan in its cell wall from host recognition. It has been demonstrated previously that β-glucan becomes exposed during infection *in vivo* but the mechanism behind this exposure was unknown. Here, we show that this unmasking involves neutrophil extracellular trap (NET) mediated attack, which triggers changes in fungal cell wall architecture that enhance immune recognition by the Dectin-1 β-glucan receptor *in vitro*. Furthermore, using a mouse model of disseminated candidiasis, we demonstrate the requirement for neutrophils in triggering these fungal cell wall changes *in vivo*. Importantly, we found that fungal epitope unmasking requires an active fungal response in addition to the stimulus provided by neutrophil attack. NET-mediated damage initiates fungal MAP kinase-driven responses, particularly by Hog1, that dynamically relocalize cell wall remodeling machinery including Chs3, Phr1 and Sur7. Neutrophil-initiated cell wall disruptions augment some macrophage cytokine responses to attacked fungi. This work provides insight into host-pathogen interactions during disseminated candidiasis, including valuable information about how the *C*. *albicans* cell wall responds to the biotic stress of immune attack. Our results highlight the important but underappreciated concept that pattern recognition during infection is dynamic and depends on the host-pathogen dialog.

## Author Summary

Opportunistic fungal infections, including those caused by *C*. *albicans*, have emerged as a significant global health burden and the disseminated form of these infections still have unacceptably high mortality rates despite modern antifungal treatments. The fungal cell wall controls its interaction with the host environment and immune recognition, although cell wall dynamics during infection are poorly understood. *C*. *albicans* organizes its cell wall to mask the inflammatory β-glucan as a form of immune evasion and it is known that during infection this β-glucan becomes exposed. Here, we investigated how β-glucan becomes exposed and discovered a dynamic interaction where host NETs provoke an active fungal response that disrupts cell wall architecture and unmasks β-glucan. We revealed an unexpected level of local fungal cell wall dynamics in response to immune mediated stress, suggesting this may represent a model that can be leveraged to identify novel drug targets. Our results highlight the understudied concept that the cell wall is a dynamic landscape during infection and can be influenced by the host.

## Introduction

Innate immune recognition of pathogen-specific patterns plays a crucial role in initial infection control and activation of appropriate adaptive immune responses [1, 2]. Recognition through Toll-like, C-type lectin, Nod-like and Rig-I-like receptors elicits production of autocrine, paracrine and endocrine immunity. This includes activities as varied as deployment of neutrophil extracellular traps to directly attack pathogens and production of proinflammatory cytokines that recruit, activate and polarize additional innate and adaptive immune cells.

Pattern recognition receptors have evolved over millions of generations, and pathogens have concurrently developed creative ways to avoid these receptors by hiding specific epitopes. Epitope masking is practiced by many pathogens including bacteria, viruses, fungi, protozoans and helminths [3–9]. Work from a number of groups, including ours, has described how fungal cell wall architecture limits recognition of the β-glucan sugar by immune receptors that include Dectin-1, a C-type lectin crucial for resistance to fungal infections [5, 6, 10]. This epitope masking can be observed in *Candida albicans*, an opportunistic human pathogen which can cause both superficial mucosal and life threatening disseminated disease, particularly in immune compromised patients. However, *C*. *albicans* β-glucan epitope availability increases dramatically *in vivo* during a phase of neutrophilic influx in experimental murine candidemia [11, 12]. Although the dynamics of immune recognition during infection have implications for the trajectory of the immune response, the fungal and host mechanisms that lead to eventual β-glucan masking *in vivo* are unknown.

It is possible that the host, the fungus or both contribute to these changes in immune recognition during infection. On the fungal side, the cell wall integrity (CWI) pathway is critical in maintaining this compartment in response to abiotic stresses, but we still don’t understand how it functions in the context of immune attack in the challenging host environment [13]. We have previously described how a highly interconnected cell wall remodeling network creates and maintains the cell wall architecture that masks β-glucan from Dectin-1 under steady-state conditions, and this network may also act *in vivo* [7]. On the host side, cell-mediated immune attack by neutrophils can kill or incapacitate pathogens using reactive oxygen and nitrogen species, antimicrobial peptides, proteases, glycosidases, and extracellular traps (ETs) [14, 15]. Proteases and glycosidases could act on the outer mannan layer to directly expose underlying β-glucan, or phagocyte attack could indirectly trigger active fungal cell wall remodeling that unmasks underlying epitopes.

Changes in *C*. *albicans* cell wall β-glucan exposure due to early host-pathogen interaction during infection may sufficiently alter availability of cell wall epitopes to affect subsequent immune responses. However, the complexity of *in vivo* systems has limited our understanding of whether immune attack regulates subsequent immune cytokine elicitation. Here, we use a combination of *in vitro* and *in vivo* tools to show that neutrophils counter β-glucan masking by creating NETs that are required to trigger fungi to actively remodel local cell wall architecture. These disruptions of cell wall epitope masking alter recognition of the fungi and could enhance subsequent secondary immune responses.

## Results

### Neutrophils disrupt cell wall organization and cause β-glucan unmasking *in vitro*

**Fig 1:**
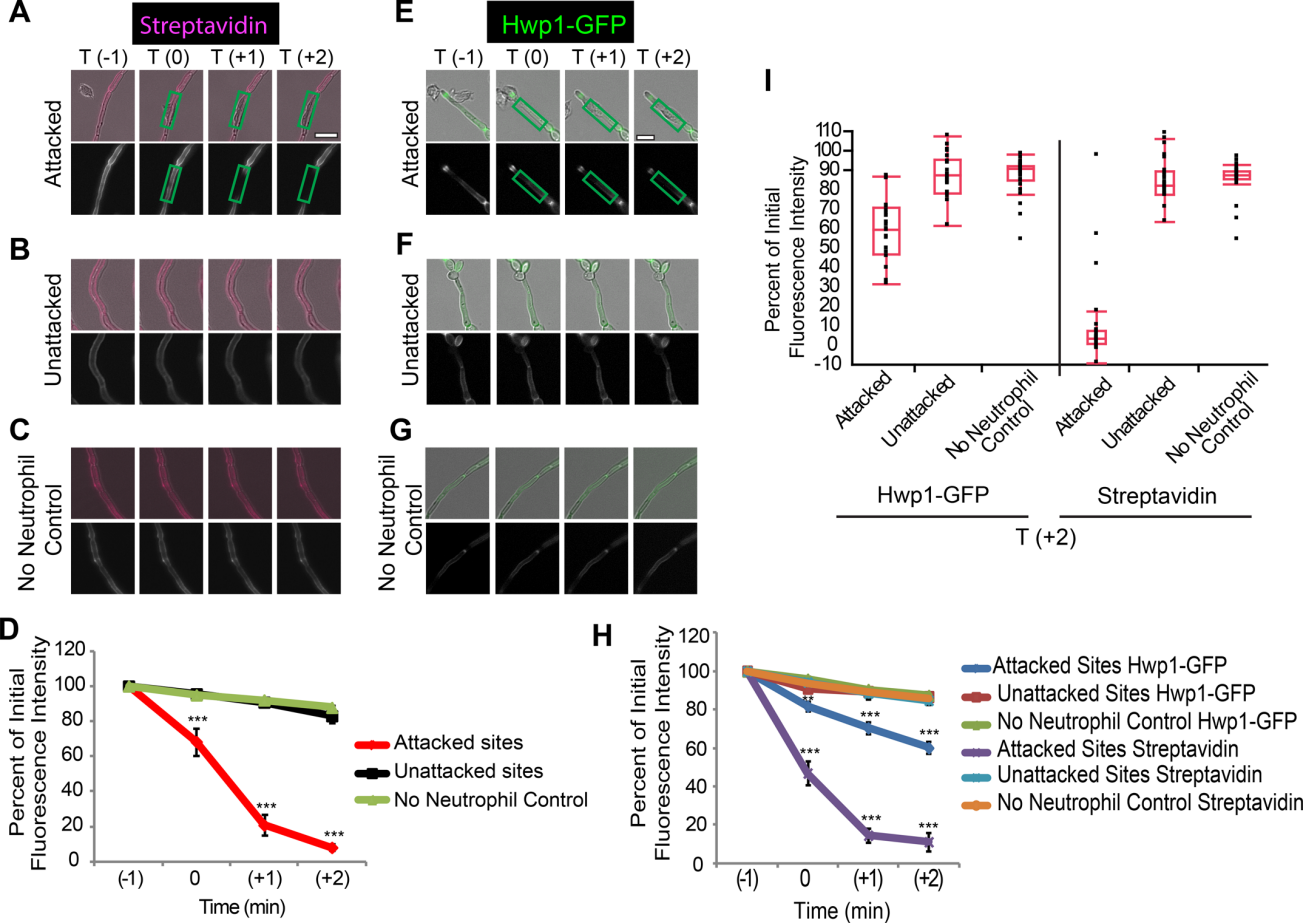
Neutrophil attachment results in rapid cell wall damage to *C*.*albicans*. (A-D) Streptavidin-Alexa 647-labeled SC5314-GFP was incubated with or without neutrophils. (A-C) Representative timelapse images at one minute intervals for (A) attacked, (B) unattacked and (C) control hyphal segments. (D) Relative streptavidin mean fluorescence intensity (MFI) at attacked and unattacked sites. Data represents the pooled average of thirteen cells measured in three experiments ± SEM. Scale bar = 10 μm. (E-I). Streptavidin-Alexa 647-labeled Hwp1-GFP fungi were incubated with or without neutrophils. (E-G) Representative time-lapse images for (E) attacked, (F) unattacked and (G) control hyphal segments. (H) Relative streptavidin intensity for twenty three cells and Hwp1-GFP intensity for thirty three cells imaged in three independent experiments was measured and the pooled average ± SEM is shown. (I) Relative intensity at the 2 minute post-attachment (T+2) time point is shown for the pooled data as box plots to illustrate the full dataset. Box plot whiskers represent the 1.5 interquartile range either below or above the lower or upper quartile ** p ≤0.01 and *** p≤0.001 (one-way ANOVA with Tukey’s post-test).

Changes to the cell wall during infection alter *C*. *albicans* recognition by pattern recognition receptors, but the mechanisms driving these changes are unknown [12, 16]. Host defense against invasive candidiasis relies critically on neutrophils, evidenced by the increased susceptibility of neutropenic patients to candidemia [17]. We reasoned that they may disrupt the fungal cell wall and mediate β-glucan unmasking because neutrophils can damage the *C*. *albicans* cell wall and are present in high numbers during infection when β-glucan unmasking appears [11, 12, 18]. To determine the spatiotemporal dynamics of neutrophilic damage, we labeled biotinylated fungi with streptavidin-Alexa 647 and incubated with neutrophils. Time-lapse imaging shows that streptavidin fluorescence is lost rapidly at sites of neutrophil attack (Fig 1A–1D, S1 and S2 Movies). Controls demonstrate that there is also a loss of labeled protein (S1 Fig). Fluorescence of an Hwp1-GFP fusion protein, present in the hyphal cell wall, is also rapidly reduced upon neutrophil attack (Fig 1E–1I, S3 and S4 Movies). Overall, this suggests that neutrophils rapidly damage cell wall protein at sites of attack, in agreement with and extending previous work [18].

**Fig 2:**
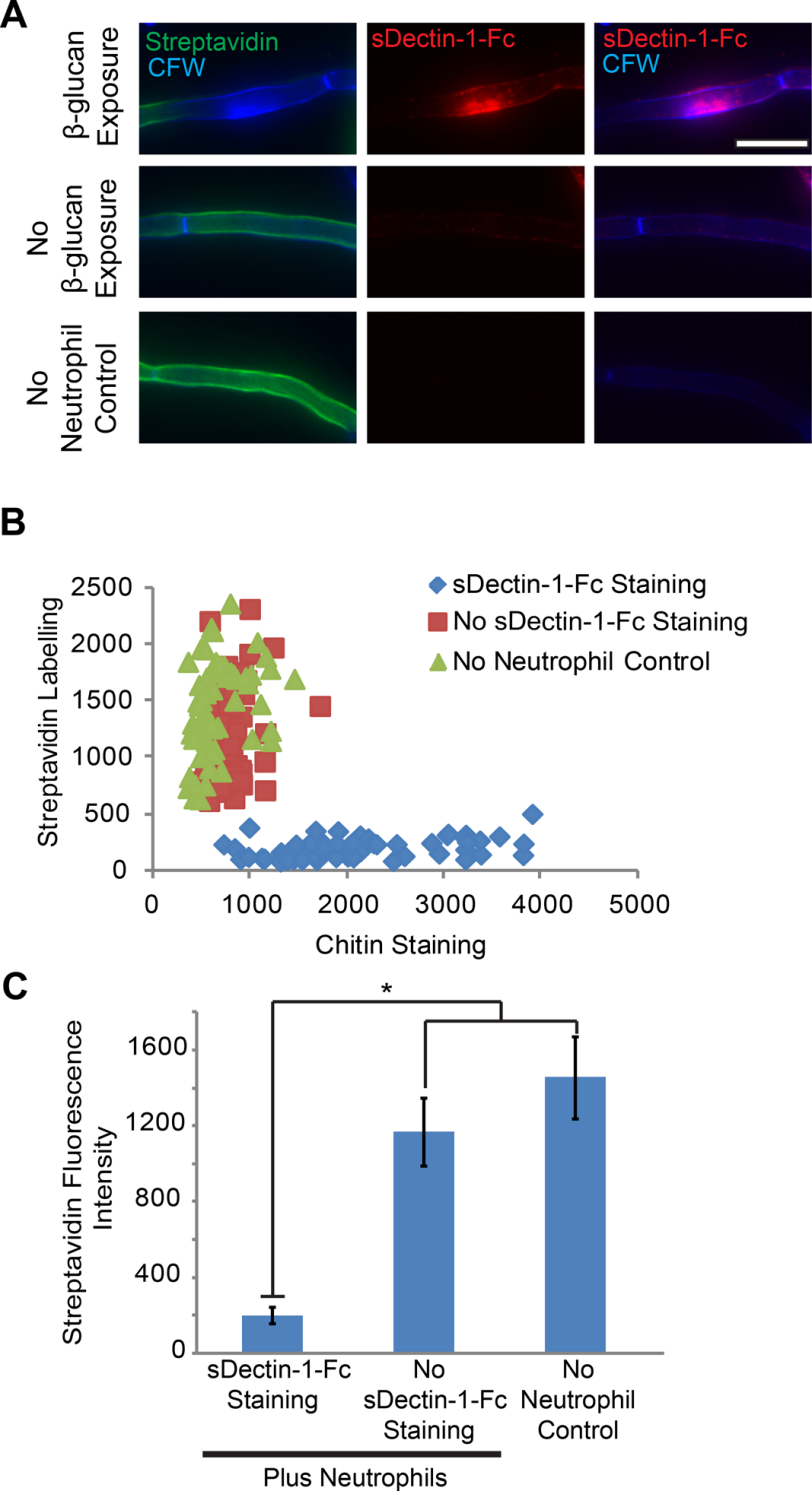
Neutrophils cause β-glucan unmasking and disrupt cell wall architecture. (A-C) SC5314-GFP cells were biotinylated, labeled with Streptavidin-Alexa647 and incubated with neutrophils or alone. Neutrophils were lysed and fungi were stained with sDectin-1-Fc and Calcofluor White. (A) A representative set of images for pre-challenge streptavidin labeling. (B-C) Images were analyzed by obtaining the mean fluorescent intensity in the blue, red and far red channels at sites of β-glucan exposure, at sites without β-glucan exposure and from sites in the no neutrophil control, limited to viable cell segments. (B) Streptavidin versus CFW fluorescence for individual sites from three pooled experiments, broken down into sites with sDectin-1-Fc staining or not. (C) Streptavidin fluorescence at sites with sDectin-1-Fc staining or no sDectin-1-Fc staining. Mean MFI ± SEM of three independent experiments. Scale bar represents 10 μm. * p ≤0.05 (one-way ANOVA with Tukey’s post-test).

To determine if neutrophil attack disrupts or triggers disruption of other aspects of cell wall architecture to alter immune recognition, we stained attacked filaments with soluble Dectin-1-Fc (sDectin-1-Fc) to assess β-glucan availability and Calcofluor white (CFW) to stain chitin. Because CFW has been shown to be a stain in live yeast cells that is selective for new chitin fibers [19], we refer to sites in the lateral wall with increased CFW staining as “Sites of chitin deposition.” This staining of attacked filaments revealed areas of the lateral cell wall with β-glucan unmasking and increased chitin deposition at sites with cell wall protein loss (Fig 2). These overlapping sites of cell wall disruption occur uniquely in the neutrophil challenged samples but not in the absence of neutrophils, demonstrating that they are a direct or indirect result of immune activity. Taken together, these results show that neutrophil attack can result in and may be an important trigger for the disruption of *C*. *albican*s’ cell wall architecture and β-glucan unmasking *in vitro*.

### Neutrophils are critical for β-glucan unmasking during disseminated candidiasis *in vivo*

**Fig 3:**
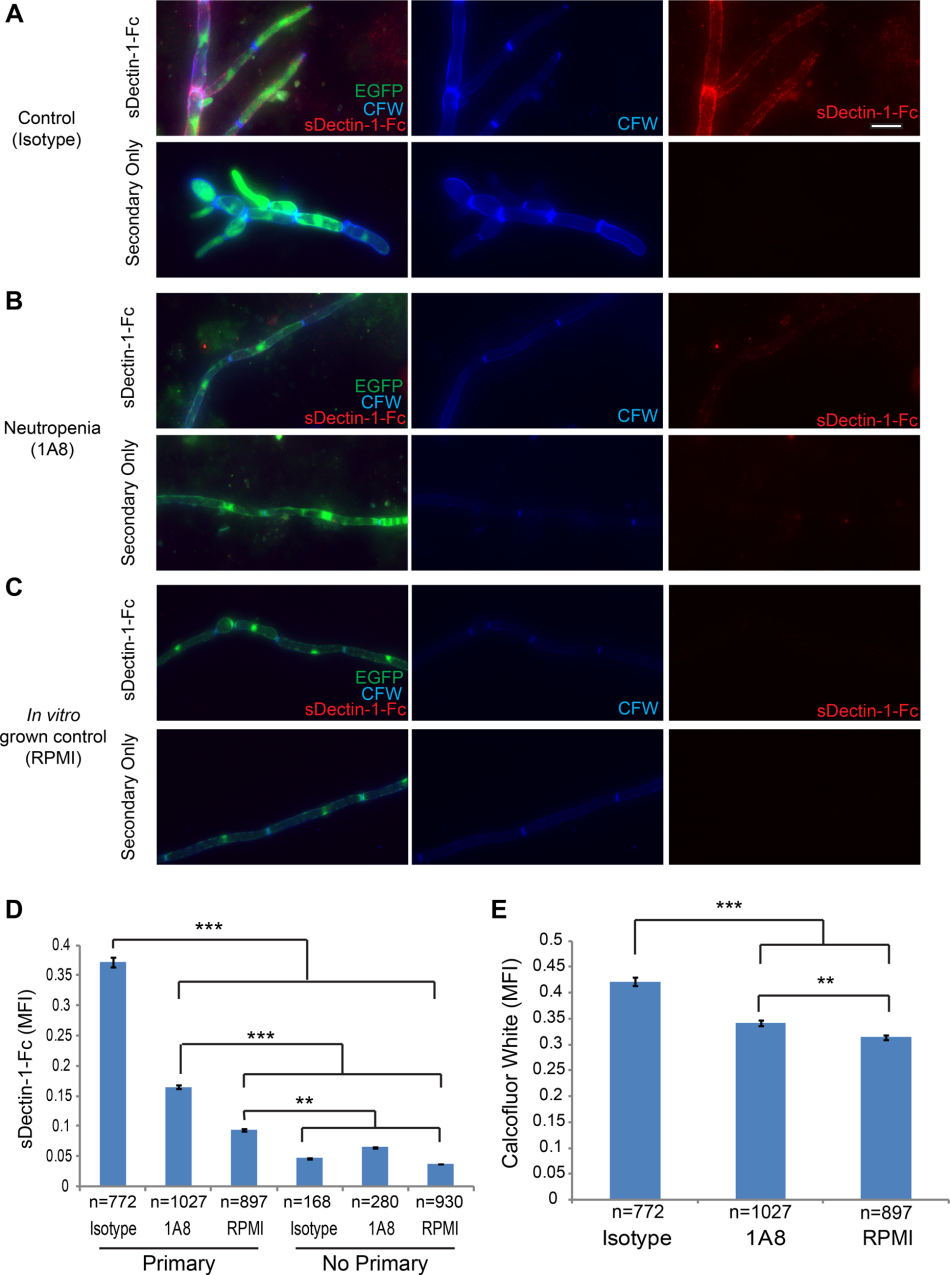
Neutrophils are critical for unmasking *C*. *albicans* β-glucan in the kidney during disseminated candidiasis. (A-E) BALB/cJ mice were injected in the tail vein with SC5314-GFP. They were then treated with either 1A8 antibody to induce neutropenia or IgG2a isotype control antibody via i.p. injection at day two post infection and sacrificed at day five post infection. (A-B) Representative images of kidney homogenates stained with sDectin-1-Fc and Calcofluor White. Bottom panels show homogenates treated with secondary antibody only as a control. (C) Representative images of an overnight culture of SC5314-GFP grown in RPMI. Scale bar represents 10 μm. (D-E) Quantification of sDectin-1-Fc staining (D) and chitin staining (E). Data is presented as the mean ± SEM from three pooled experiments. ** p-value ≤0.01 and *** p-value ≤0.001 (Kruskal-Wallis with Dunn’s post-test).

We have previously shown β-glucan unmasking occurs during infection and our *in vitro* data suggests that neutrophils can mediate this exposure [12]. To test if neutrophils are required for these fungal cell wall changes *in vivo*, we examined *C*. *albicans* epitope exposure in neutropenic mice at day 5 post-infection, when there is normally β-glucan unmasking. To interrogate the native state of the *C*. *albicans* cell surface we used the *ex vivo* fluorescence method, which involves no fixation or permeabilization [12]. There is a significant reduction in β-glucan unmasking in neutropenic mice, demonstrating that neutrophils are critical for β-glucan unmasking *in vivo* (Fig 3A–3D). Similar results are seen in a second model of neutropenia, and as expected both methods of inducing neutropenia also led to increased susceptibility to infection (S2 Fig). Levels of β-glucan unmasking were similar when detected via either anti-β-glucan antibody or sDectin-1-Fc staining, demonstrating that this is not an artifact of a specific probe (S2 Fig). Further, chitin staining revealed that fungi from control mice have significantly stronger chitin deposition than neutropenic mice, suggesting that neutrophil attack is also important for increased chitin deposition *in vivo* (Fig 3A–3C and 3E). Fungi from neutropenic mice have slightly increased β-glucan unmasking and chitin levels as compared to *in vitro* RPMI-grown control cells, suggesting that growth in the host or possibly attack by other immune cells may also yield minor but significant cell wall changes even without neutrophil attack. Taken together, these results demonstrate that neutrophils are critical drivers of β-glucan unmasking and increased chitin deposition during disseminated infection *in vivo*.

### NET attack results in fungal cell wall disruption

**Fig 4:**
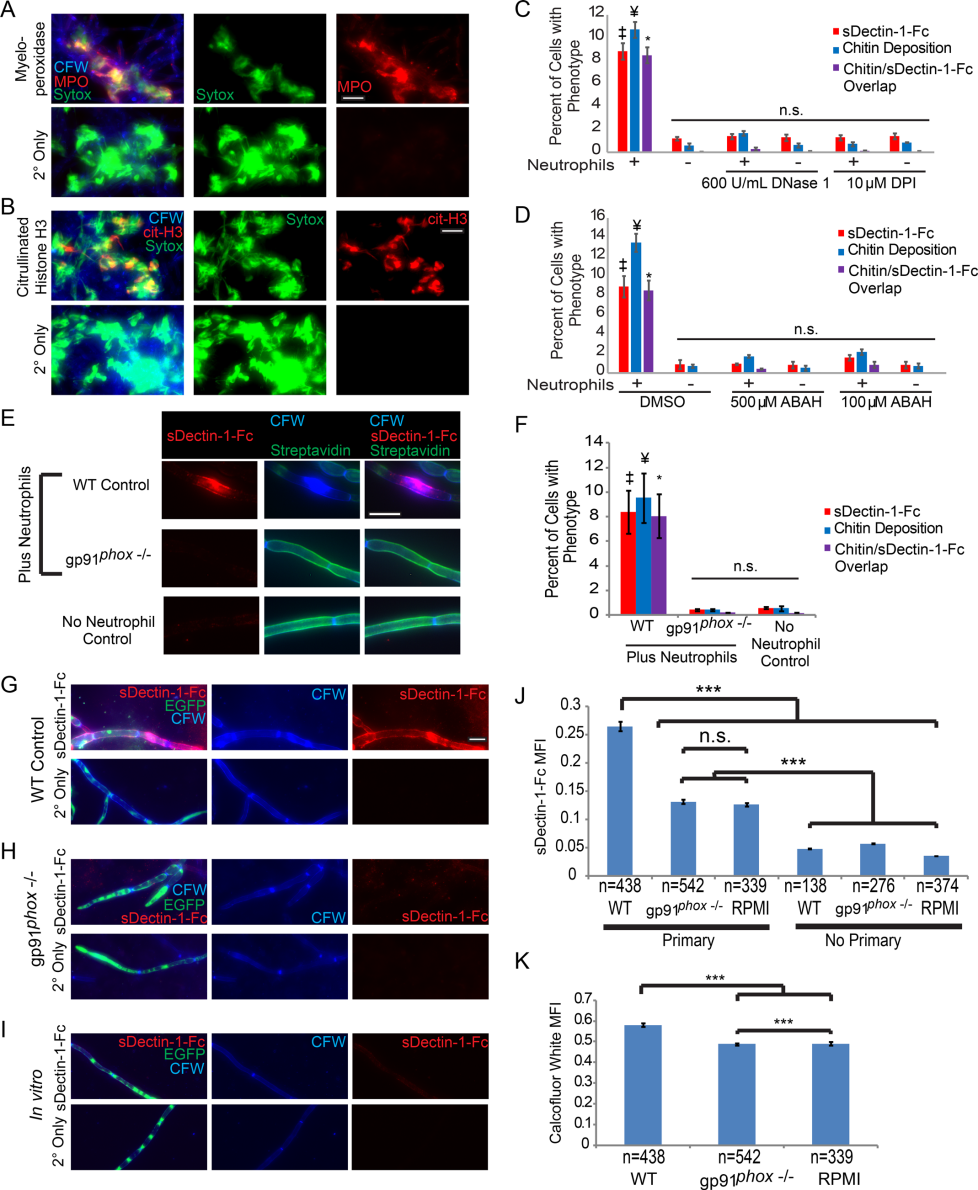
NETs are critical for immune attack to cause β-glucan unmasking. (A-B) Neutrophils from C57BL/6J mice were incubated with WT-FarRed670 hyphae. Neutrophils were not lysed and samples were stained for MPO or citrullinated H3. Representative images of MPO (A) and citrullinated H3 staining (B). Scale bar represents 20 μm. (C-D) Neutrophils were pretreated with DNase I, DPI or ABAH and then incubated with WT-FarRed670 for 2.5 hours. Neutrophils were not lysed and then samples were stained with CFW, sDectin-1-Fc and Sytox Green. Images were analyzed by scoring all viable cell segments for the presence of the indicated phenotypes and results are presented as the percentage of total cell segments counted which displayed the phenotype for each group. Data is presented as the mean ± SEM from three experiments. Cell viability was determined using characteristic cytoplasmic far red expression (see Methods). ‡ p-value ≤ 0.01 when comparing the sDectin-1-Fc group from WT or DMSO treated to other groups. ¥ p-value ≤ 0.01 when comparing the chitin deposition group from WT or DMSO treated to other groups. * p-value ≤ 0.01 when comparing the overlap group from WT or DMSO treated to other groups. Comparisons done with one way ANOVA and Tukey’s post-test. (E-F) Neutrophils from C57BL/6J or gp91^phox-/-^ mice were incubated with Streptavidin-Alexa 647 labelled SC5314-GFP hyphae. Neutrophils were lysed and fungi were stained with sDectin-1-Fc and Calcofluor White. (E) A representative set of images are shown for each group. (F) Images were analyzed by scoring all viable cell segments for the presence of the indicated phenotypes and results are presented as the percentage of total cell segments counted which displayed the phenotype for each group. Data is presented as the mean ± SEM from three experiments. Cell viability was determined using characteristic cytoplasmic GFP expression (see Methods). ‡ p-value ≤ 0.01 when comparing the sDectin-1-Fc group from WT to either gp91^phox-/-^ or no neutrophil groups. ¥ p-value ≤ 0.01 when comparing the chitin deposition group from WT to either gp91^phox-/-^ or no neutrophil groups. * p-value ≤ 0.01 when comparing the overlap group from WT to either gp91^phox-/-^ or no neutrophil groups. Comparisons done with one way ANOVA and Tukey’s post-test. (G-K) C57BL/6J or gp91^phox-/-^ mice were injected in the tail vein with SC5314-GFP and kidneys were harvested on day 5 post infection. (G-H) Representative images of kidney homogenates stained with sDectin-1-Fc and CFW Bottom panels show homogenates treated with secondary antibody only as a control. (I) Representative images of an overnight culture of SC5314-GFP grown in RPMI and then stained with sDectin-1-Fc and CFW. (J-K) Quantification of sDectin-1-Fc staining (J) and chitin staining (K). Data is presented as the mean ± SEM from two pooled experiments, except for the RPMI group which represents a single experiment. *** p-value ≤0.001 (Kruskal-Wallis with Dunn’s post-test). n.s means non-significant. Scale bar represents 10 μm.

It was not previously known that neutrophils alter innate pattern recognition of fungi *in vivo*, so we sought to characterize the mechanisms required to alter epitope unmasking. NET production, in which neutrophils create traps out of DNA and numerous antimicrobial factors, is a means of neutrophil attack against *C*. *albicans* and other fungi *in vivo* and *in vitro* [20–24]. Despite the poor NET production of mouse neutrophils relative to human neutrophils, we find strong evidence of NET formation *in vitro*. These NETs stain positive with the membrane impermeant Sytox green DNA dye, anti-citrullinated histone antibody, and anti-myeloperoxidase (MPO) antibody (Fig 4A and 4B). Furthermore, we observed that neutrophils could rapidly create ETs on C. albicans hyphae (S5 and S6 Movies, S3 Fig). Strikingly, treatment with DNase I to degrade extracellular DNA and prevent the establishment of NETs blocks both chitin deposition and β-glucan unmasking, functionally implicating NETs in driving this interaction (Fig 4C). In further support of NET-triggered changes during attack, inhibition of myeloperoxidase (MPO) with 4-aminobenzoic acid hydrazide (ABAH) prevents neutrophil attack from resulting in chitin deposition or β-glucan unmasking (Fig 4D). Taken together, these data provide strong evidence that NETs provide the initial stimulus that results in fungal cell wall changes including β-glucan exposure.

Neutrophil proteases are thought to be an important component of NET formation in some contexts and neutrophil elastase trafficking is regulated during NETosis against *C*. *albicans* [20, 25, 26]. However, neutrophils from mice deficient in the dipeptidyl peptidase (DPPI), which is required for the activation of the three major neutrophil proteases: elastase, cathepsin G and proteinase 3 [27], show no defect in their ability to cause β-glucan unmasking, chitin deposition or streptavidin loss (S4 Fig).Thus, these three proteases do not appear to play an important role in the downstream cell wall remodeling triggered by neutrophil attack of *C*. *albicans* in this system.

Phagocyte NADPH oxidase is important in defense against candidemia and plays an important role in NET formation under many conditions, including in response to fungi [20, 28, 29]. We find that disruption of NADPH oxidase function, either by using neutrophils from gp91^phox-/-^ mice lacking a key component of the NADPH oxidase or using chemical inhibitors, decreases cell wall damage and prevents immune attack from resulting in β-glucan unmasking or chitin deposition *in vitro* (S5 Fig, Fig 4C, 4E and 4F). This was not due to a complete lack of neutrophil attack on the hyphae (S5 Fig). Similarly, fungi from the kidneys of gp91^*phox-/-*^ mice had significantly less chitin staining and β-glucan unmasking, demonstrating the importance of phagocyte oxidase for causing cell wall remodeling *in vivo* (Fig 4G–4K). As expected, WT mice were able to control fungal growth while gp91^*phox-/-*^ mice were unable to do so (S5 Fig). Importantly, immune cells including many neutrophils were found surrounding hyphae in gp91^*phox-/-*^ mice (S5 Fig), suggesting that the loss of β-glucan unmasking was not due to lack of immune cell recruitment. Taken together, these data suggest that NETs also trigger *C*. *albicans* cell wall remodeling and enhanced Dectin-1 recognition *in vivo* and reveal a new way that immune cells counter fungal immune evasion.

### Chitin deposition and β-glucan unmasking are an active fungal response to neutrophil attack

**Fig 5:**
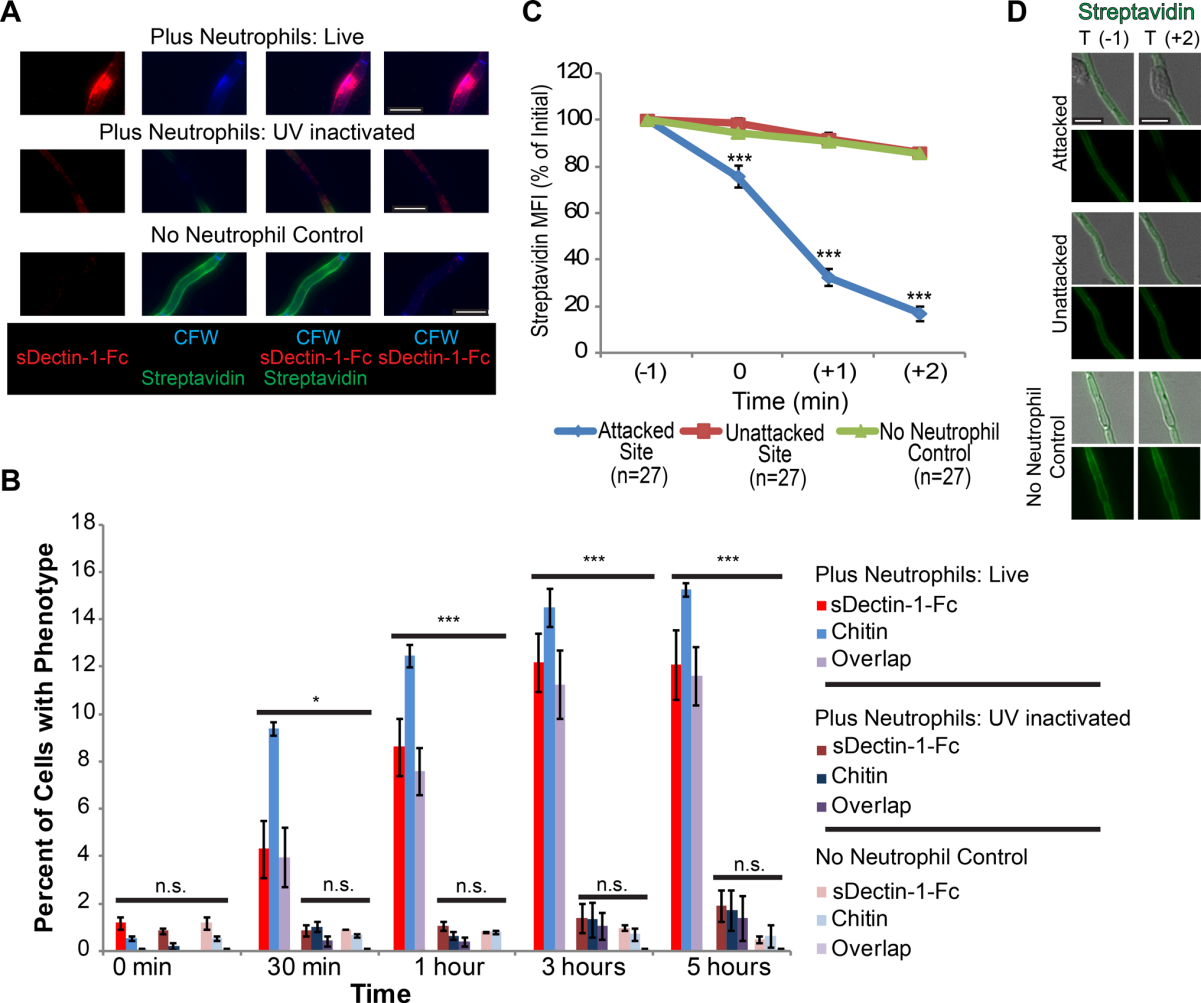
Chitin deposition and β-glucan unmasking result from an active fungal process. (A-B) Streptavidin-Alexa 647 labeled SC5314-GFP hyphae were either UV inactivated or not before being incubated with neutrophils or alone for the amount of time indicated. (A) Representative images of cells from each group at the three hour timepoint are shown. (B) Quantitation of cells for the phenotype indicated over the timecourse. Cells were scored for the indicated phenotype and results are presented as the percent of total cells for each time point. The data represents the mean ± SEM of three independent experiments. (C-D) Streptavidin-Alexa 647-labeled SC5314-GFP was UV inactivated and incubated with or without neutrophils. (C) Results represent the pooled average MFI at sites from individual frames in timelapses, as in Fig 1D. (D) Representative images of the T(-1) and T(+2) timepoints from timelapses of UV inactivated *C*. *albicans*. Scale bars represent 10 μm. * p-value of < 0.05 and *** p-value of ≤ 0.001 (one way ANOVA with Tukey’s post-test). n.s. means non-significant.

Although NET attack could directly cause these cell wall changes, NET damage could also initiate conserved fungal stress signaling pathways that are known to both respond to cell wall insults and mask β-glucan in steady-state [7, 30]. We reasoned that if neutrophil-triggered changes are passive from the fungal perspective, they should occur rapidly and simultaneously, and should also occur in inactivated fungi. Surprisingly, although initial cell wall protein damage occurs within seconds (Fig 1), chitin deposition is not apparent until 30 minutes post-challenge, and enhanced Dectin-1 recognition lags even further (Fig 5A and 5B). A similiar succession of events also occurs in experiments conducted in imaging dishes (S6 Fig).

The nature of these sequential changes over hours suggests that unmasking results from an active fungal response rather than by direct immune mediated damage. In support of this hypothesis, UV-inactivated fungi lose streptavidin at attack sites but fail to develop sites of chitin deposition or β-glucan unmasking (Fig 5). UV inactivation is a minimally invasive means of killing fungi, so these results indicate that only initial cell wall damage is a direct result of immune attack (Fig 5C and 5D, S7 and S8 Movies). Thus, it appears that immune attack triggers β-glucan unmasking and chitin deposition only indirectly, by promoting active fungal signaling in response to immune mediated attack.

### The fungal response to neutrophil attack includes cell wall integrity signaling and remodeling

**Fig 6:**
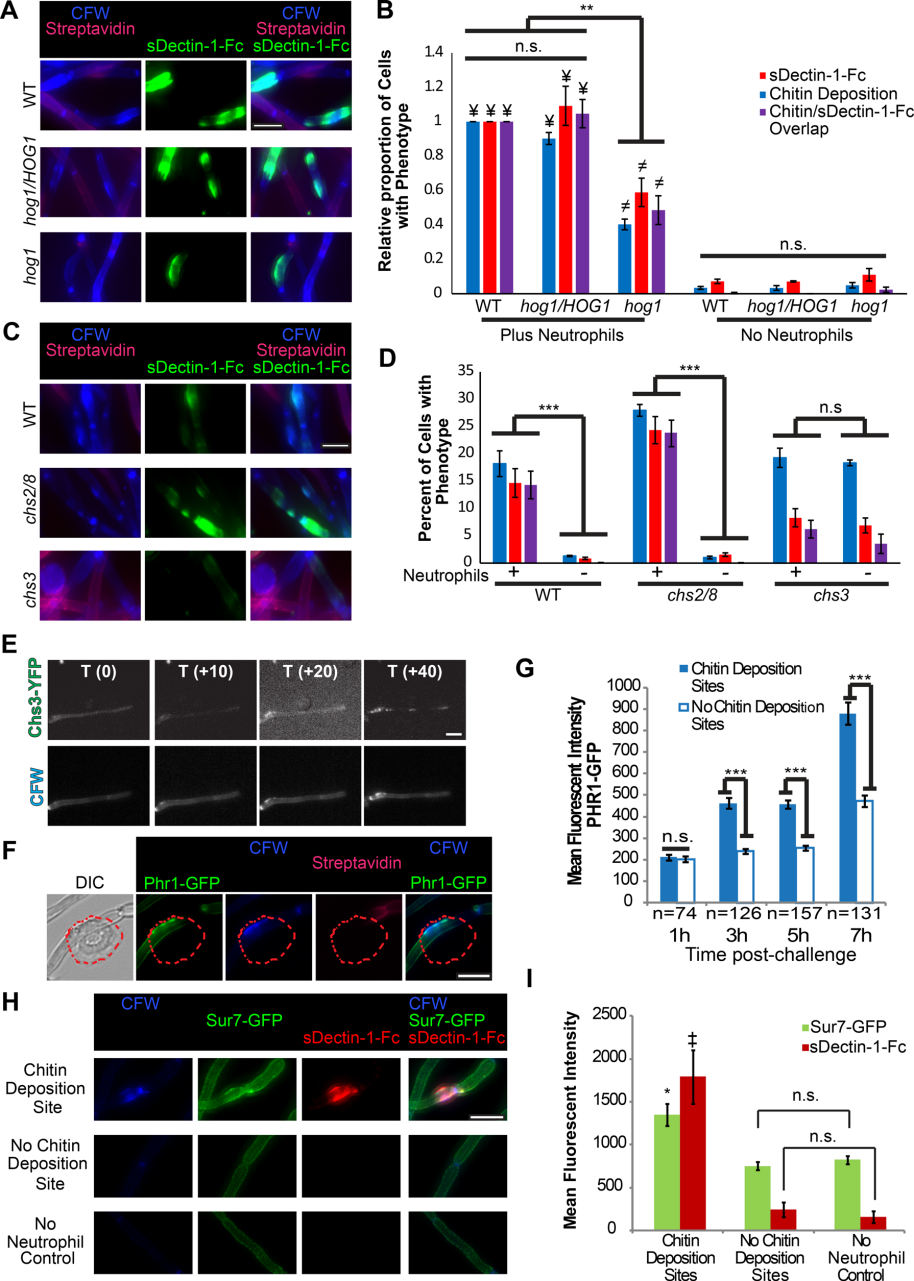
Cell wall integrity sensing and remodeling are involved in the fungal response to neutrophil attack. (A-D) Streptavidin-Alexa 647 labeled *C*. *albicans* hyphae of the indicated strains were incubated with neutrophils or alone. Neutrophils were not lysed and samples were stained with CFW and sDectin-1-Fc. (A) Representative images of the *HOG1* strain set. Images were analyzed by scoring cells for localized chitin deposition, sDectin-1-Fc staining and the overlap of the two phenotypes. (B) The scoring data was normalized based on the frequency of cell wall changes in the wildtype strain plus neutrophils and represents the mean ± SEM from three independent experiments. (C) Representative images of the chitin synthase strain set. Images were analyzed by scoring cells for increased chitin deposition, sDectin-1-Fc staining and the overlap of the two phenotypes. (D) Data is presented as the percent of total cells with the phenotypes and represents the mean ± SEM from three independent experiments. The Chs3-YFP strain was incubated with neutrophils in an imaging dish and timelapses were taken. (E) Panels taken from a timelapse of the Chs3-YFP strain after neutrophil attack. (F) The JC94-2 strain was incubated with neutrophils for the indicated time in an imaging dish before being imaged. Representative images from the 3 hour timepoint. The red dotted line represents the outline of a neutrophil. (G) Images were analyzed by obtaining the mean fluorescent intensity of GFP at sites with or without chitin deposition and the data is presented as the mean MFI ± SEM at sites from three pooled experiments except the 1h timepoint, which represents two pooled experiments. (H-I) The Sur7-GFP strain was incubated with neutrophils. Following incubation, neutrophils were lysed and fungi were stained with sDectin-1-Fc and CFW. Data represents the MFI of GFP or Cy3 staining at sites with or without chitin deposition or from the no neutrophil control and is presented as the mean MFI ± SEM from three pooled experiments. * p-value of ≤0.05, ** p-value of ≤0.01 and *** p-value of ≤0.001. For I, * p≤0.05 comparing Sur7-GFP at chitin deposition sites vs no chitin deposition or no PMN control. (¥) p≤0.001 comparing the WT and *hog1/HOG1* plus neutrophil groups to the no neutrophil control groups. (≠) p≤ 0.01 comparing the *hog1* plus neutrophils group to the no neutrophil control groups. (‡) p≤ 0.01 comparing sDectin-1-Fc at chitin deposition sites vs no chitin deposition sites or no neutrophil controls. (Student’s T-test for D and G or one way ANOVA with Tukey’s post-test for B and I). n.s. means non-significant. Scale bar represents 10 μm.

The fungal cell wall integrity (CWI) signaling pathway plays a key role in stress responses and in maintaining the normal cell wall architecture that masks β-glucan [7, 30]. However, it is not known how *C*. *albicans* responds to immune-mediated cell wall damage, so we sought to identify which signaling pathway(s) drives localized cell wall remodeling. Targeted screening of mutants deficient in individual CWI signaling components for defects in responding to neutrophil attack revealed that *HOG1* is important for this process while *CEK1* and *MKC1* are not required (Fig 6A and 6B, S7 Fig). The *HOG1*-deficient strain has a significantly decreased ability to respond to neutrophil attack with both chitin deposition and β-glucan unmasking (Fig 6A and 6B, S7 Fig). This defect is not due to differences in fungal cell viability or attack rates between strains (S7 Fig, S8 Fig). Interestingly, *C*. *albicans* deficient in *CAP1*, which is involved in responding to some types of oxidative stress [31, 32], is not required for this chitin deposition response (S8 Fig). This primary dependence on Hog1p suggests that chitin deposition and enhanced Dectin-1 binding result from post-transcriptional activities, as Hog1p plays a limited role in regulating stress-mediated transcription [13]. It is important to note that, while deficient in its responses, the residual responses of the *hog1* strain suggest other pathways are also involved.

Increased chitin levels can rescue *C*. *albicans* from stress, including antifungal treatment [33]. To implicate a specific synthase in enhanced chitin deposition at attack sites, we examined post-attack chitin deposition in mutants in either the major chitin synthase, *CHS3*, or both stress-activated synthases *CHS2* and *CHS8* [34]. Both WT and *chs2*∆/∆ *chs8*∆/∆ strains have dramatic increases in areas with localized chitin deposition and β-glucan unmasking following interaction with neutrophils when compared to their no neutrophil controls (Fig 6C and 6D). However, while the abnormal morphology of the *chs3*∆/∆ deletion mutant results in a high baseline number of areas with increased chitin deposition and to a lesser extent β-glucan exposure, there is no significant increase in cell wall changes after neutrophil attack. This defect was not due to differences in cell viability, number of attacked sites, or lack of cell wall damage (S8 Fig). Analysis of the intensity of chitin staining is consistent with a major role for Chs3p in driving neutrophil-triggered chitin deposition (S8 Fig). The *chs3*∆/∆ mutant was not completely deficient in responding to attack with chitin deposition, however, suggesting that other chitin synthases may play a very limited role in this process. In support of the idea that Chs3p is the major synthase in the response to neutrophil damage, timelapse of a Chs3-YFP fusion strain demonstrates recruitment of Chs3p-YFP to most sites of increased chitin deposition (Fig 6E, S9 Movie).

Cell wall remodeling is also crucial for response to stress, but we know little about the spatiotemporal dynamics of these responses, especially in the context of immune attack. We therefore characterized the post-attack movement of cell wall remodeling and biogenesis proteins, including Sur7p and Phr1p. Sur7p is deposited in new cell wall and marks eisosomes, and Phr1p is a β(1,3)-glucan remodeling enzyme crucial to cell wall integrity [35, 36]. Both Phr1p and Sur7p are recruited to sites of neutrophil attack. Sur7p was recruited early and coincident with chitin deposition while Phr1p accumulated at later times (Fig 6F–6I, S10 and S11 Movies). Overall, these results help elucidate important components of the fungal response to immune cell attack, suggesting Hog1p is important for the initial signaling response which leads to chitin deposition mainly through Chs3p localization and the later cell wall remodeling possibly involving Phr1p and Sur7p.

### Neutrophil-mediated cell wall disruption can enhance immune responses

**Fig 7:**
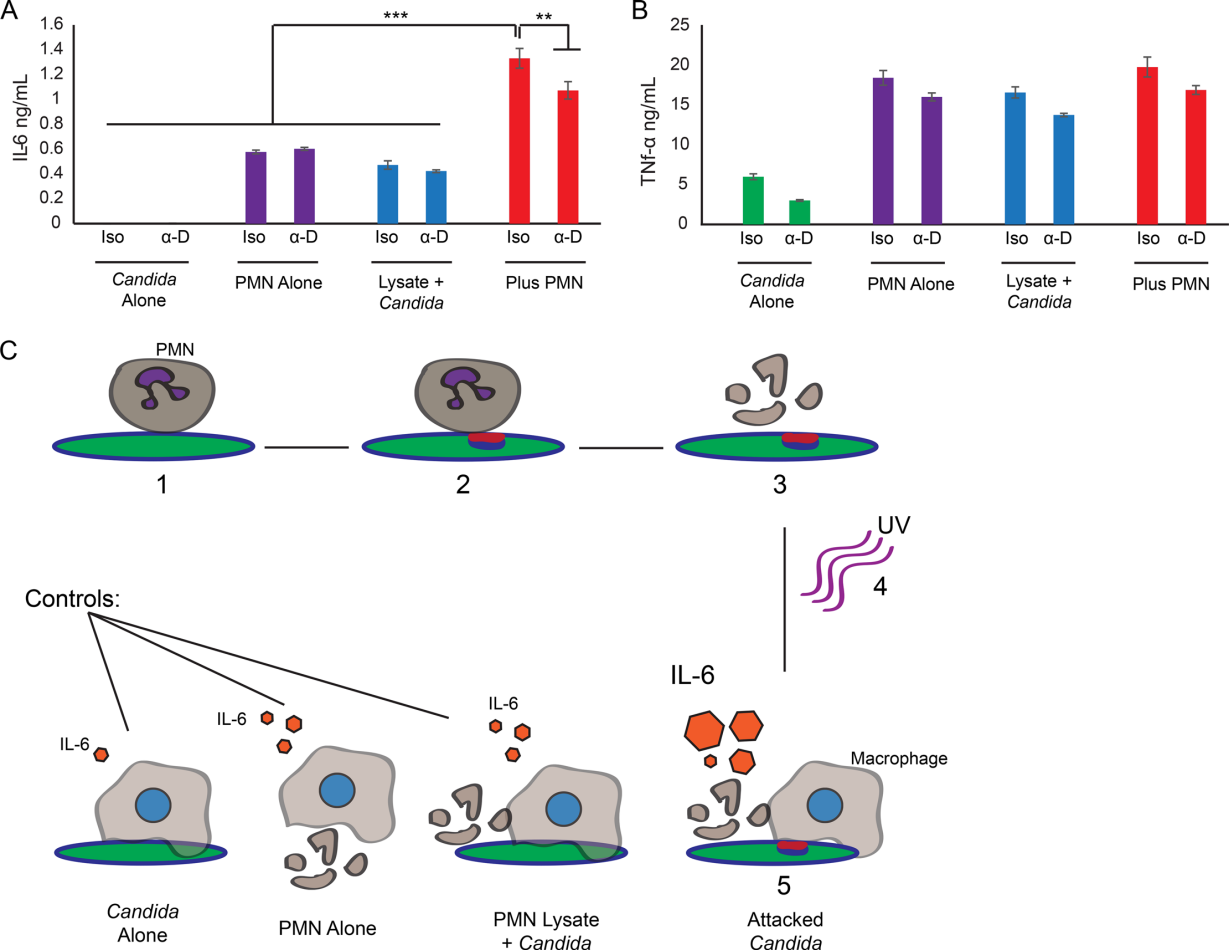
*C*. *albicans* induces an enhanced IL-6 response after neutrophil attack. RAW-blue cells were treated as indicated and incubated for 6 hours. (A-B) ELISA was conducted on supernatants from RAW-Blue cells to detect (A) IL-6 or (B) TNF-α. A representative experiment is shown for each cytokine. Comparisons were done by two way ANOVA with Tukey’s post-test. ** p-value of ≤0.01 and *** p-value of ≤0.001. (C) Schematic representation of the macrophage experiment. 1.) Neutrophils are incubated with *C*. *albicans* overnight. 2.) During this incubation, neutrophils attack *C*. *albicans*, initiating cell wall remodeling and resulting in β-glucan unmasking and chitin deposition. 3.) Following incubation, neutrophils are lysed and samples are treated with DNase 1 to reduce activation by neutrophil debris. 4.) Samples are UV inactivated. 5.) Samples are then incubated with macrophages. Macrophages recognize the exposed fungal epitopes and produce cytokines in response. The attacked fungi with increased epitope unmasking elicit more IL-6 in comparison to the controls.

Neutrophil-triggered enhancement of Dectin-1 binding may result in an altered secondary immune response to *C*. *albicans* or have no impact due to redundant recognition modalities. To assay secondary immune responses, we challenged *C*. *albicans* hyphae with neutrophils, then lysed the neutrophils. We treated the samples with DNase1 to reduce activation of macrophages by neutrophil debris before UV-inactivating the fungi and adding them to murine macrophages. A mixture of unattacked fungi with neutrophil lysate served as a control for activation by remaining neutrophil debris in the context of fungal stimulation [37]. ELISA assays revealed that attacked fungi induced higher production of the proinflammatory cytokine IL-6 when compared to any of several controls (Fig 7). Interestingly, this increased cytokine production was not completely dampened by Dectin-1 inhibition suggesting other receptors may also be involved in this response. These results indicate that neutrophil attack and the resulting cell wall changes, including β-glucan unmasking, can lead to enhanced recognition and responses by other immune cells.

## Discussion

Recognition of pathogens based on conserved molecular patterns is a cornerstone of innate immunity but it is a dynamic battlefield between host and pathogen. The host’s task is complicated because pathogens conceal essential molecular patterns from detection, thereby denying the host the knowledge it needs to initiate a response. Here, we build on previous work to show that although *C*. *albicans* masks β-glucan during infection, host immune cells can damage the invader and trigger the disruption of cell wall architecture in a manner that could enhance innate immune recognition, including the unmasking of β-glucan. Our findings echo work in diverse animal and plant hosts that suggest pathogen recognition and responses are dynamic and can impact immunity during infection [16, 38–40]. Other types of “unmasking” might take place in a number of infections, as masking of epitopes has been demonstrated for bacteria [3], viruses [4], fungi [5–7], protozoans [8] and helminths [9]. While the microbial cell wall is an adaptable landscape that is capable of responding to numerous stimuli, we still have a limited understanding of how cell wall architecture changes *in vivo* and how host-pathogen interactions influence PAMP availability during infection.

We describe here how the host subverts a fungal evasion strategy, unmasking *C*. *albicans* to reveal fungal-specific epitopes like β-glucan. Surprisingly, this is a two-step process where NET-dependent neutrophil attack results in β-glucan unmasking via an active fungal process. The fungal response to localized cell wall stress includes a cascade of events, with chitin deposition and enhanced β-glucan exposure mediated by Hog1p signaling and the major chitin synthase Chs3p. Remodeling of cell wall architecture enhances recognition and could enhance responses by the host, but is also likely to protect the fungus by strengthening the cell wall, as is the case for plant cell walls that remodel upon fungal attack [41, 42]. These mechanisms of cell wall architecture control during fungal infection are likely relevant for other fungi that hide immunogenic β-glucan from the host and thereby limit immune responses [5, 6, 43]. Given the importance of Dectin-1 signaling in anti-fungal defense, and the fact that NETs are deployed against other fungi, it seems likely that immune mediated unmasking may take place during other fungal infections [21, 44, 45].

The ability of a host to recognize and respond to microbe-specific components is a key determinant to mounting an effective defense. Neutrophil unmasking of hyphal β-glucan, which is structurally distinct and elicits greater inflammatory cytokine responses than yeast β-glucan, could help the host discriminate between commensalism and opportunistic disease [46], especially since *C*. *albicans* hyphae are typically associated with invasion and greater recognition could enhance the “danger” response to invading hyphae [47]. Unmasked epitopes could also assist in “trained immunity” for other innate immune cells like monocytes, which has been shown to depend on fungal β-glucan and Dectin-1 signaling for protection against *C*. *albicans* [48]. Our results demonstrate that cell wall changes following neutrophil attack do increase recognition of β-glucan and also specifically elicit more IL-6, but not TNF-α, from macrophages when compared to controls. Elevated IL-6 production, in particular, may be important as it participates in the induction of Th17 responses which are important for antifungal immunity [49–51]. Interestingly, some of the increased macrophage response was not blocked by neutralizing anti-Dectin-1 antibodies, suggesting that either other cell wall changes beyond β-glucan unmasking may play a role or the antibody is less effective in our system. In addition to enhanced Dectin-1 recognition, it is likely that post-attack disruption of cell wall architecture results in alterations to other fungal cell wall epitopes. Our preliminary data using wheat germ agglutinin as a probe suggests there is also increased availability of chitin at attack sites. Because fungal chitin recognition can modulate inflammation, altered chitin recognition may contribute to secondary immune responses [52, 53].

The impact these epitope changes might have on the outcome of infection remains to be explored. While elevated IL-6 could contribute to the development of protective Th17 response after recognition of exposed fungal epitopes, hyperinflammation of the IL-17 axis and excessive neutrophilic influx are also risk factors, and recent work suggests that myeloid-derived suppressor cell-mediated immunomodulation is protective during this phase around 4–7 days post-infection [54–58], [59]. Whether for protection or pathogenesis, the potential of immune attack to alter subsequent immune response suggests that immune dynamics may play an important regulatory role.

Extracellular traps function in pathogen containment and killing, and we find that they can also influence pathogen epitope exposure. The specific requirements for neutrophils and phagocyte oxidase to damage *C*. *albicans* and initiate unmasking *in vivo* fits with previous *in vitro* findings that ETs made by macrophages don’t damage *Candida* [60] and our own preliminary observations that macrophage attack doesn’t elicit the same changes to the *C*. *albicans* cell wall. NADPH oxidase and MPO are known players in NET formation [61], but NET formation has also been shown to occur in a reactive oxygen species independent manner so it is still unknown if or how these components contribute to the fungal damage that induces cell wall remodeling. While some NETs were still produced upon NADPH oxidase inhibition, our data demonstrates the requirement for functional NADPH oxidase, MPO and extracellular DNA in NETs for inducing fungal cell wall changes following immune attack. This suggests that the role of the NADPH oxidase and MPO is not primarily in NET creation but instead may contribute to decorating NETs with damaging components which provoke fungal integrity responses (with the role of the NETs themselves to hold these components in close proximity to the fungal cell wall). It is possible that MPO activity alone could provoke fungal cell wall changes but the inhibitory nature of DNAse1 treatment combined with the requirement of NADPH oxidase all point to NETs playing a key role. Further experiments will be required to fully understand the exact role NETs and their components are playing. The MPO requirement suggests this is not strictly due to the neutrophil respiratory burst causing localized hypoxia, which is an environmental condition previously associated with fungal cell wall changes [62]. Surprisingly, our experiments suggest that neutrophil proteases are not required for NET-dependent unmasking in our system, although they have been previously implicated in human and mouse NET formation [20, 25, 26]. We tested this by using neutrophils deficient in DPPI, in which the three major neutrophil proteases could not be processed properly [27]. Interestingly, it has also been seen that DPPI deficiency limits PMA and ROS-induced NET production [26]. As more research emerges, the requirement for different components in NET production has been found to be highly context and stimulus dependent, even for elements like the phagocyte NAPDH oxidase which were previously thought to be absolutely critical [29, 61]. It is therefore possible that we have identified a situation in which these proteases do not play a critical role in NET formation or that the defects which result are not severe enough to compromise their function in the unmasking process. Further work will be required to determine if this lack of a requirement is because other proteases can fill in during this situation or if no protease activity is required at all.

The early initiation of NET-dependent *C*. *albicans* cell wall remodeling, by 30 minutes post-challenge, suggests a deployment of NETs within thirty minutes—much earlier than previous reports of NETosis attack of *C*. *albicans* [20, 24, 63]. Rapid NET formation has been previously described to occur in a subset of neutrophils in response to certain stimuli [25]. It is notable that the relatively early NET formation we describe here as triggering fungal cell wall remodeling is dependent on NADPH oxidase activity, in contrast to “live” NETosis that has been previously described as NADPH oxidase-independent [64]. The mechanism of NET release here is unknown and remains to be analyzed. A better understanding of neutrophil and NET function during infection could have clinical benefits, as defects in either result in increased susceptibility to many infections, including those caused by *Candida* [17, 65].

The mechanism whereby neutrophil attack reveals fungal epitopes is unexpected, as cell wall changes are not a direct result of immune attack but rather are initiated by signaling in the fungus. The importance of the Hog1p MAPK in response to neutrophil attack is consistent with its established roles in interactions with phagocytes and host immunity both *in vitro* and *in vivo* [66, 67]. While Hog1p clearly plays an important role, the *hog1Δ/Δ* strain is not completely deficient in responding to neutrophil attack, suggesting that other pathways can play a minor compensatory role. The lack of a requirement for *CEK1* and *MKC1* is intriguing, as they both play important roles in cell wall homeostasis and stress adaptation [30, 68]. This suggests that the importance of each CWI MAPK is context-dependent and specific for given set of stress factor(s). Considering previous reports, our data offer the possibility that *hog1*Δ/Δ hypersensitivity to neutrophil-mediated killing may be due to a failure to deposit chitin and reinforce its cell wall, a process that rescues *C*. *albicans* from other stresses [33]. This role for Hog1p adds to its previously described activities in regulation of osmotic and oxidative stress [69].

The localized cell wall stress caused by neutrophil attack provides an advantageous situation to dynamically model how *C*. *albicans* hyphae mobilize their cell wall machinery in response to neutrophil attack *in vivo*. Genetic deletion mutants show that Chs3p is responsible for the majority of this localized lateral cell wall chitin deposition and is important for eventual β-glucan unmasking, while Chs2p and Chs8p are not required for these changes. Time-lapse microscopy also demonstrated accumulation of Chs3-YFP at attack sites with chitin deposition, suggesting a new role for Chs3p in responding to neutrophil-mediated stress. The requirement of chitin synthases may be context dependent, as recent reports show Chs2p and Chs8p are involved in maintaining cellular integrity during some forms of stress *in vitro* [70]. This data, combined with that showing the importance of Hog1p signaling in responding with chitin deposition, supports previous observations that Hog1p can regulate and activate chitin synthesis [71]. The recruitment of cell wall regulatory and remodeling enzymes Sur7p and Phr1p suggests a multistep process of remodeling post-attack. While Phr1p is known to be recruited to apical growth sites and septa and to respond to other stresses, this is the first time it has been found enriched in a localized section of lateral cell wall [36, 72]. Sur7p is important in regulating cell wall organization and integrity [35]. Early Sur7p enrichment at sites of chitin deposition and β-glucan unmasking suggests it plays an early role in cell wall reorganization at sites of neutrophil attack, in contrast with the later role for Phr1p. Beyond providing detailed insight into how *C*. *albicans* responds to neutrophil attack, this model of localized cell wall stress offers a powerful new method to image the multistep dynamics of stress-stimulated cell wall remodeling.

β-glucan recognition is relevant beyond mammalian immunity, as it has been demonstrated that both invertebrates and plants sense and respond to β-glucan, with important implications for antifungal immunity [73, 74]. Indeed, dynamic host-pathogen interactions revolving around fungal β-glucan masking and host recognition also occur during infections in plants, suggesting parallels with important agricultural fungal infections [39, 41, 75].

The game of pathogen camouflage and host-mediated unmasking has been played out over generations throughout the animal, fungal and plant kingdoms. The localized fungal cell wall remodeling we observe upon immune-mediated stress represents a novel model to probe basic mechanisms of cell wall dynamics and may identify novel therapeutic targets or strategies especially relevant to the *in vivo* infection environment.

## Materials and Methods

### *C*. *albicans* strains and growth conditions

*C*. *albicans* strains used in this study are listed in the Table 1. *C*. *albicans* was maintained on YPD 37°C. Single colonies were picked to 5 mL YPD liquid and grown at 30°C overnight on a rotator wheel. For hyphal cells, a defined number of yeast cells were transferred into RPMI and grown in 5 mL tubes at 30°C overnight on a rotator wheel. JC94-2 was constructed from the JC94 parent [36]. *PHR1-GFP*, along with 1kb upstream and 0.5 kb downstream regulatory sequence, was amplified from JC94 genomic DNA with primers PHR1-CIp20-FOR (5’-ATATTCGACTGA**AAGCTT**GATTACAAGTGGGATGCAAAA-3’), and PHR1-CIp20-REV (5’-TCGTCGGGCTCA**AAGCTT**CGTTGAAAAAGCATAAGAAGG-3’) and cloned into CIp20 [76] with HinDIII, after which it was sequence verified. Integration of Stu1-cut CIp20-PHR1-GFP at the RP10 locus was confirmed by PCR and three independent clones had similar phenotypes.

**Table 1:** Strain details.

Strain names used	Parental Strain	Source or reference	Genotype
SC5314-GFP	SC5314	[12]	*Peno1*::*Peno1*-*EGFP-NAT* ^R^
*hog1*∆/∆-dTom	JC50	[77]	*ura3*:: λ *imm434/ura3*::*limm434*, *his1*::*hisG*/*his1*::*hisG*, *hog1*::l*oxP-ura3-loxP*, *hog1*::*loxP-HIS-loxP* CIp20 (*URA3*, *HIS1*), *Peno1*::*Peno1-dTom-NAT* ^R^
*hog1/HOG1*-dTom	JC52	[77]	*ura3*:: λ *imm434/ura3*::*limm434*, *his1*::*hisG*/*his1*::*hisG*, *hog1*::*loxP-ura3-loxP*, *hog1*::l*oxP-HIS-loxP* CIp20-*HOG1* (*URA3*, *HIS1*), *Peno1*::*Peno1-dTom-NAT* ^R^
WT-dTom (for *hog1* strains)	JC21	[77]	*ura3*:: λ *imm434/ura3*::*limm434*, *his1*::*hisG*/*his1*::*hisG* CIp20 (*URA3*, *HIS1*), *Peno1*::*Peno1-dTom-NAT* ^R^
*cap1/CAP1*-dTom	JC807	[31]	*cap1*::*loxP-HIS1-loxP/cap1*::*loxP-ARG4-loxP*,CIp20-*CAP1* (*URA3*, *HIS1*), *Peno1*::*Peno1-dTom-NAT* ^R^
WT-dTom (for *cap1* strains)	JC747	[78]	SN148 + CIp30 (*URA3 HIS1 ARG4*), *Peno1*::*Peno1-dTom-NAT* ^R^
*cap1*∆/∆-dTom	JC842	[78]	*cap1*::*loxP-HIS1-loxP/cap1*::*loxP-ARG4-loxP*, CIp20 (URA3), *Peno1*::*Peno1-dTom-NAT* ^R^
Phr1-GFP/JC94	CAF3-1	[36]	*PHR1/PHR1-GFP ura3Δ*::*imm434/ura3Δ*::*imm434*
JC94-2	JC-94	This work	*PHR1/PHR1-GFP ura3Δ*::*imm434/ura3Δ*::*imm434I*, *RPS1/RPS1*::CIp20-*PHR1-GFP*
CAI4-dTom	CAI-4	[79]	Δ*ura3*::*imm434*/Δ*ura3*::*imm434*, *Peno1*::*Peno1-dTom-NAT* ^R^
*chs3*∆/∆-dTom	*chs3*∆/∆	[80]	*chs3*Δ::*hisG/chs3*Δ::*hisG*, *Peno1*::*Peno1-dTom-NAT* ^R^
*chs2*∆/∆ *chs8*∆/∆ -dTom	NGY138	[81]	*chs2*Δ::*hisG/chs2*Δ::*hisG*, *chs8*Δ::*hisG*/*chs8*Δ::*hisG*, *Peno1*::*Peno1-dTom-NAT* ^R^
SN250		[82]	*his1*∆/*his1*∆, *leu2*∆::*C*.*dublinensis HIS1*/*leu2*∆::*C*.*maltosa LEU2*, *arg4*∆/*arg4*∆, *URA3*/*ura3*∆::*imm* ^*434*^, *IRO1*/*iro1*∆::*imm* ^*434*^
*cek1*∆/∆-SN	SN250	[82]	*his1*∆/*his1*∆, *leu2*∆/*leu2*∆, *arg4*∆/*arg4*∆, *URA3*/*ura3*∆::*imm* ^*434*^, *IRO1*/*iro1*∆::*imm* ^*434*^, *cek1*∆::*C*.*dublinensis HIS1*/*cek1*∆::*C*.*maltosa LEU2*
*mkc1*∆/∆-SN	SN250	[82]	*his1*∆/*his1*∆, *leu2*∆/*leu2*∆, *arg4*∆/*arg4*∆, *URA3*/*ura3*∆::*imm* ^*434*^, *IRO1*/*iro1*∆::*imm* ^*434*^, *mkc1*∆::*C*.*dublinensis HIS1*/*mkc1*∆::*C*.*maltosa LEU2*
*hog1*∆/∆-SN	SN250	[82]	*his1*∆/*his1*∆, *leu2*∆/*leu2*∆, *arg4*∆/*arg4*∆, *URA3*/*ura3*∆::*imm* ^*434*^, *IRO1*/*iro1*∆::*imm* ^*434*^, *hog1*∆::*C*.*dublinensis HIS1*/*hog1*∆::*C*.*maltosa LEU2*
Sur7-GFP	YHXW4	[83]	*ura3*Δ::λ*imm434/ura3*Δ::λ*imm434 his1*::*hisG/his1*::*hisG arg4*::*hisG/arg4*::*hisG SUR7-GFPγ*::*URA3*
Hwp1-GFP	YJB8250	[84]	*ura3*::λ*imm434/ura3*::λ*imm434 his1*::*hisG/his1*::*hisG arg4*::*hisG/arg4*::*hisG Hwp1/Hwp1-GFP*
WT-FarRed670	SC5314	This work	*Peno1*::*Peno1*-*FarRed670-NAT* ^R^
Chs3-YFP	NGY477	[34]	*ura3*::*imm434/ura3*::*imm434*, *his1*::*hisG*/*his1*::*hisG*, *arg4*::*hisG/arg4*::*hisG*, *CHS3/CHS3–YFP*:*URA3*, *RPS1/RPS1*::CIp30

### Incubation of *C*. *albicans* and neutrophils *ex vivo*

Neutrophils from 6–12 week old female C57BL/6J or gp91^phox-/-^ (B6.129S-*Cybb* ^*tm1Din*^/J (Jackson Laboratories) [85]) mice were purified from bone marrow using biotin anti-Ly6G antibody (eBioscience) and AutoMACS separation (Miltenyi). Bone marrow from sex- and age-matched C57BL/6-backcrossed DPPI -/- and control mice was extracted after overnight shipment on cold packs [86]. *C*. *albicans* hyphae at a concentration of 3x10^8^ cells/mL were labeled with Biotin-XX-SSE (Molecular Probes; 0.01 μg/μL). Cells were then labeled with Alexa Fluor 647-conjugated Streptavidin (Jackson Immunoresearch; 36 μg/mL). 3 x10^7^ hyphal cells were then incubated with or without 7 x 10^6^ neutrophils in RPMI + 5% FBS for 2.5 hours. Neutrophils were lysed with 0.02% Triton X-100, though all samples also received this treatment. *C*. *albicans* hyphae were then stained with sDectin-1-Fc (17 μg/ml) followed by donkey anti-human IgG Cy3 or Alexa Fluor 488 antibody (Jackson Immunoresearch; 0.8 mg/ml) and Calcofluor White (Sigma Chemicals; 25 ng/mL). Purified sDectin-1-Fc was prepared from stably transfected HEK293T cells as previously described [87]. Cells were visualized by optical sectioning fluorescence microscopy using a Zeiss Axiovision Vivotome microscope (Carl Zeiss Microscopy, LLC). Fields of view were chosen randomly and an equal number of images were obtained for each sample. Maximum image projections were used to score the percentage of live cells with increased chitin deposition, β-glucan exposure and the overlap of both phenotypes. The tips of hyphae were excluded from scoring to prevent confusion by any cell wall changes occurring during hyphal growth. For some experiments, cells were also scored for the absence of streptavidin labeling. Cells from the “no neutrophils” samples had a baseline level of cells without streptavidin labeling of approximately 20% due to growth post-labeling, while samples with neutrophils had little to no additional growth over the course of the experiment and therefore had a low baseline level of cells without streptavidin labeling. Cell viability was confirmed based on the characteristic cytoplasmic EGFP, dTom or FarRed670 expression of live cells, or by propidium iodide (250 ng/mL) exclusion in non-transformed strains.

For post-challenge labeling experiments, hyphae were biotinylated, and incubated with neutrophils; after neutrophil lysis and staining, Alexa Fluor 647-conjugated Streptavidin was included along with the secondary antibody. For chemical inhibition, neutrophils were pre-incubated with either the vehicle DMSO, 10 μM DPI, 300 μM Apocynin, 100 μM ABAH or 500 μM ABAH for 10 minutes before addition to *C*. *albicans* and samples were treated for the entire 2.5 hours. For UV inactivation experiments, hyphal cells were UV inactivated as described [7]. To ensure that lack of damage was not due to altered neutrophil attack rates, staining in experiments with the *hog1*Δ/Δ, *cap1*Δ/Δ, *cek1*Δ/Δ, *mkc1*Δ/Δ, *chs2*Δ/Δ *chs8*Δ/Δ and *chs3*Δ/Δ mutant strains was performed without neutrophil lysis with Triton X-100 treatment. To examine NETs, sDectin-1-Fc and CFW staining procedures were carried out as described above, with Sytox Green (Molecular Probes; 156 nM) added along with the secondary antibody and CFW. We used Anti-Histone H3 citrulline R2+R8+R17 (abcam; 0.014 mg/mL) and donkey anti-rabbit IgG Cy3 (Jackson Immunoresearch; 0.0075 mg/mL) as well as Anti-MPO (R&D Systems; 0.1 mg/mL) with Donkey anti-goat Cy3 (Jackson Immunoresearch; 0.007 mg/mL). In experiments with DNase 1, the RPMI used for the incubation was supplemented with 100 mM CaCl_2_ and 100 mM MgCl_2_.

### Imaging dish experiments

Streptavidin-labeled hyphae of the indicated strain, at a concentration of 6x10^6^ cells, were added to a Delta T imaging dish (Bioptechs Inc) with 8x10^5^ neutrophils in 1 mL of Phenol red-free RPMI + 5%FBS (Lonza). The Chs3-YFP strain was not labeled and imaged in 1 mL of PBS with 5% FBS and 5.5 mM glucose. Imaging dishes were then either incubated at 37°C in an incubator for the indicated amount of time or immediately imaged on the Zeiss Axiovision Vivotome microscope (Carl Zeiss Microscopy, LLC) or Nikon PerfectFocus microscope (Nikon) with a heated stage (Bioptechs, Inc) at 37°C. Chs3-YFP timelapses were instead taken on a Nikon Ti-E PFS live cell microscope (Nikon, Inc). For staining in dishes, the sDectin-1-Fc and CFW staining was done as described in the above section except 4.25x10^6^ neutrophils were added, they were not lysed and the process was carried out in the dish.

### *In vivo* infections and organ *ex vivo* fluorescence

Six week old female Balb/cJ, C57BL/6J or gp91^phox-/-^ mice (Jackson Laboratories) were infected via tail vein. WT, isotype controls (Rat IgG2a for 1A8 from Bio X cell) and RB6-8C5 (Bio X cell) treated mice received 1x10^5^ cfu, those receiving 1A8 received 2.5x10^4^ cfu and gp91^phox-/-^ mice received 500 cfu. Mice were treated with isotype, RB6-8C5 or 1A8 antibody (Bio X cell; 100 μg in 200 μL of PBS) via i.p. injection on day 2 post-infection. On day 5 post-infection mice were sacrificed via CO_2_ inhalation followed by cervical dislocation. Neutropenia was confirmed by Wright staining of blood obtained by cardiac puncture. Organs were harvested and homogenized as described [12]. For some experiments, kidneys were bisected with a razor and half was processed for histology. Homogenates were stained with sDectin-1-Fc (17 μg/ml) then donkey anti-human IgG Cy3 (0.8 mg/ml) and Calcofluor White (25 ng/mL). Alternatively, homogenates were stained with anti-β-glucan antibody (Biosupplies, Inc., Australia; 1.7 mg/mL) then with goat anti-mouse Cy3 antibody (Jackson Immunoresearch; 3.8 mg/mL). Cells were visualized by optical sectioning fluorescence microscopy using a Zeiss Axiovision Vivotome microscope (Carl Zeiss Microscopy, LLC). Maximum projection images were quantified using Cellprofiler (www.cellprofiler.org) as described [12].

### Macrophage cytokine elicitation

RAW-blue macrophages (Invivogen) were maintained in DMEM + 10% FBS supplemented with sodium pyruvate, gentamicin and zeocin. For detection of cytokines by ELISA, unlabeled 3.0x10^7^ *C*. *albicans* hyphae were incubated with or without 7x10^6^ neutrophils overnight in RPMI+5%FBS. A neutrophil alone group was also included. The next morning RAW-blue macrophages were harvested. Macrophages were resuspended at 2.77x10^6^ cells/mL and pre-incubated with Anti-mDectin-1 (Bio-Rad; 10 μg) or IgG2b isotype control (Invivogen; 10 μg) for 90 minutes. *Candida*-neutrophil mixtures were treated with 0.05% Triton X-100 solution for 5 minutes. As control for the impact of neutrophil debris in the context of fungal stimulation, one of the neutrophil alone samples was added to the *C*. *albicans* alone sample just before lysis. Samples were washed extensively and then incubated with 200 units of DNase1 for 1 hour. Samples were then washed extensively again and resuspended in 500 μL PBS. 25 μL of sample was added to the wells of a 96 well plate in duplicate before UV inactivation by treatment with 5 x 100,000 μJ/cm^2^. For a positive control, depleted zymosan (Invivogen) was added and for a negative control sterile water was added. RAW-blue cells, either Anti-mDectin-1 treated or not, were then added to the UV-inactivated fungal cells at 5x10^5^ cells per well. Supernatants were harvested after 6 hours. ELISA was performed using Mouse TNF-α and IL-6 DuoSets (R&D Systems) according to manufacturer’s instructions and detected using Supersignal ELISA Femto Maximum Sensitivity Substrate (ThermoFisher Scientific) using a Biotek Synergy 2 plate reader (Biotek Instruments, Inc).

### Statistical analysis

Statistics were performed as described in figure legends. For normally distributed data, Student’s t test or one or two way ANOVA analysis with Tukey’s post-test were used. For non-parametric data, Kruskal-Wallis with Dunn’s post-test was applied. ANOVA and Kruskal-Wallis were done using Prism software (Graphpad Software). p < 0.05 was considered significant.

### Ethics statement

All animal studies were carried out in accordance with the recommendations in the Guide for the Care and Use of Laboratory Animals of the National Institutes of Health. All animals were treated in a humane manner according to guidelines of the University of Maine IACUC as detailed in protocol number A2014-02-01. The UMaine IACUC/Ethics Committee approved this protocol. Animals were euthanized by carbon dioxide inhalation. Infected animals were monitored twice daily for signs of infection and morbid animals were euthanized.

## Supporting Information

S1 Fig Neutrophil attack results in cell wall damage and protein loss. (A-B) Streptavidin-HRP labeled SC5314-GFP was incubated with neutrophils or alone. Neutrophils were lysed and fungi were then stained with an anti-HRP Cy3 antibody and CFW. Representative images for each group are shown (A). Images were analysed by obtaining the mean fluorescent intensity at sites with and without chitin deposition and data is presented as the mean ± SEM of three independent experiments (B). (C-E) SC5314-GFP was pre-labeled with biotin and then incubated with neutrophils. After incubation, neutrophils were lysed and hyphae were stained with Streptavidin-A647, sDectin-1-fc and CFW. Representative images are shown (C). Images were analyzed by obtaining the mean fluorescent intensity in the blue, red and far red channels at sites of β-glucan exposure, at sites without β-glucan exposure and from sites in the no neutrophil control, limited to viable cell segments. (D) Streptavidin fluorescence at sites with sDectin-1-Fc staining or no sDectin-1-Fc staining. Mean MFI ± SEM of three independent experiments. (E) Streptavidin versus CFW fluorescence for individual sites from three pooled experiments, broken down into sites with sDectin-1-Fc staining or not. Cell viability was confirmed based on the characteristic cytoplasmic EGFP expression of live cells (see Methods). * p ≤0.05 ** p-value ≤0.01 (one way ANOVA with Tukey’s post-test). Scale bar represents 10 μm.
(EPS)

S2 Fig Neutrophils are critical for β-glucan exposure and control of *C*. *albicans* in the kidney. BALB/cJ mice were injected in the tail vein with SC5314-GFP and were treated with either PBS or RB6-8C5 antibody via i.p. injection before being sacrificed at day five post infection. (A) Representative images of serial kidney sections from infected mice treated with PBS (A) or RB6-8C5 (B) after being stained with either hematoxylin and eosin (upper images) or Periodic acid schiff (lower images). (C-D) Representative images of kidney homogenates stained with sDectin-1-Fc. Bottom panels show homogenates treated with seconday antibody only. (E) Images were quantified by scoring *Candida* cell segments for β-glucan exposure (either exposed or non-exposed). Total number of cells found in each category were presented according to mouse treatment group. Three independent experiments were performed. A significant association between PBS treatment and exposed cells and between RB6-8C5 treatment and non-exposed cells was seen, p-value<0.0001 (Fisher’s Exact test). (F-G) Representative images of kidney homogenates stained with anti-1,3-β-glucan antibody. Bottom panels show homogenates treated with secondary antibody only. (H) Images were quantified by scoring as described for (E). A significant association between PBS treatment and exposed cells and between RB6-8C5 treatment and non-exposed cells was seen, p-value<0.0001 (Fisher’s Exact test). Scale bar represents 10 μm. (I-J) Representative images of serial kidney sections from infected mice treated with IgG2a isotype (I) or 1A8 (J) after staining with hematoxylin and eosin or periodic acid-Schiff. The scale bar in the first column of each panel represents 200 μm and in the second column represents 100 μm.
(PDF)

S3 Fig Rapid extracellular trap deployment by neutrophils. (A-B) WT-FarRed670 hyphae were incubated with neutrophils in an imaging dish with CFW and Sytox Green. Timelapses were obtained using binning 2x2 and 10 minute intervals. (A) Individual panels from the timelapse showing neutrophil ET deployment. (B) Image taken after the end of the timelapse of the area of ET deployment. Scale bar represents 5 μm.
(EPS)

S4 Fig The major neutrophil proteases are not required for *C*. *albicans* cell wall disruption. Neutrophils from WT age and sex matched controls or DPPI KO mice were incubated with Streptavidin-Alexa 647 labelled SC5314-GFP hyphae. Neutrophils were lysed and the fungi were stained with sDectin-1-Fc and Calcofluor White. (A) Representative set of images for each group. (B) Images were analyzed by scoring all viable cell segments for the presence of the indicated phenotype and the results are presented as the percentage of total cell segments counted. Data is presented as the mean ± SEM from three experiments. Cell viability was determined using characteristic cytoplasmic GFP expression (see Methods). ‡ p-value ≤ 0.05 for comparing the sDectin-1-Fc group from WT or DPPI KO to the no neutrophil group. ¥ p-value ≤ 0.05 for comparing the chitin deposition group from WT or DPPI KO to the no neutrophil group. * p-value ≤ 0.05 for comparing the overlap group from WT or DPPI KO to the no neutrophil group. Comparisons done by one way ANOVA with Tukey’s post-test. (C-F) Streptavidin-Alexa 647 labeled hyphae were incubated with either WT or DPPI KO neutrophils in an imaging dish for 30 minutes. (C-D) Representative images for each group. (E) The percent of neutrophil sites with streptavidin degradation for each group. Data represents the mean ± SEM from three independent experiments. (F) The average MFI in the far red channel at sites of neutrophil attachment for each group. Data represents the mean ± SEM from three pooled experiments. *** p-value ≤ 0.001 by one way ANOVA with Tukey’s post-test. n.s. means non-significant by Student’s Ttest (E) or one way ANOVA with Tukey’s post-test (B,F). Scale bar represents 10 μm.
(EPS)

S5 Fig Phagocyte NADPH oxidase is important for fungal cell wall disruption. (A-D) Streptavidin-Alexa 647 labeled SC5314-GFP was incubated with neutrophils or alone. Neutrophils were pre-incubated with 10 μM DPI, 300 μM Apocynin or empty vehicle (DMSO) for 10 minutes before being added to fungi. Following incubation, neutrophils were lysed and fungi were stained with sDectin-1-Fc and CFW. (A-B) Representative images are shown. (C-D) Images were analyzed by scoring viable cells for the phenotype indicated and data is presented as the percent of total cells with the mean ± SEM for three independent experiments. ‡ p-value ≤ 0.01 for comparing the sDectin-1-Fc group from DMSO to the 10 μM DPI, 300 μM Apocynin or either no neutrophil groups. ¥ p-value ≤ 0.01 for comparing the chitin deposition group from DMSO to the 10 μM DPI, 300 μM Apocynin or either no neutrophil groups. * p-value ≤ 0.01 for comparing the overlap group from DMSO to the 10 μM DPI, 300 μM Apocynin or either no neutrophil groups. n.s. means non-significant. Comparisons done by one way ANOVA with Tukey’s post-test. (E-I) Streptavidin-Alexa 647 labeled SC5314-GFP was incubated with either C57BL/6J or gp91^phox-/-^ neutrophils in an imaging dish for 30 minutes. (E-F) Representative images for each group. (G) The percent of neutrophil sites with degradation for each group. Data represents the mean ± SEM from three independent experiments, with between 130 and 275 sites scored per experiment. (H) The average MFI in the far red channel at sites of neutrophil attachment. Data represents the mean ± SEM from three pooled experiments where n represents the number of neutrophil attachment sites where MFI was measured. (I) The average MFI for each group, including a no neutrophil control, from a single experiment. (J-K) Representative images of serial kidney sections from either C57BL/6J (J) or gp91^phox-/-^ mice (K) after staining with hematoxylin and eosin or periodic acid-Schiff. The scale bar in the first column of each panel represents 200 μm and in the second column represents 100 μm. (K) Images were examined by a pathologist and example areas containing neutrophils are highlighted with red arrows. * p-value ≤ 0.05, ** p-value ≤ 0.01 and *** p-value ≤0.001 by Student’s Ttest (G) or one way ANOVA with Tukey’s post-test (H). Scale bar represents 10 μm.
(PDF)

S6 Fig Cell wall disruptions are associated with neutrophil attack. (A-B) Live *C*. *albicans* was incubated with neutrophils for the indicated time in an imaging dish before being stained and imaged. (A) Cells were scored for the indicated phenotypes and data was presented as the mean ± SEM for three experiments. (B) Representative images from the 3 hour timepoint showing cell wall changes in an area of neutrophil attachment. Scale bar represents 10 μm.
(EPS)

S7 Fig Hog1 is the major MAPK pathway involved in cell wall remodeling after neutrophil attack. (A-C) Streptavidin-Alexa 647 labeled *C*. *albicans* hyphae (WT = SN250, *cek1* = *cek1*∆/∆-SN, *mkc1* = *mkc1*∆/∆-SN, *hog1* = *hog1*∆/∆-SN; all from the collection of Noble et al. [82]) were incubated with neutrophils or alone. Neutrophils were not lysed and samples were stained with CFW, sDectin-1-Fc and propidium iodide. (A) Images were analyzed by scoring live cells for localized enhanced Calcofluor white staining, sDectin-1-Fc staining and the overlap of the two phenotypes. The scoring data was normalized based on the frequency of cell wall changes in the wildtype strain plus neutrophils and represents the mean ± SEM from three independent experiments. (B-C) Images were also analyzed by scoring all cells for (B) the absence of streptavidin labeling and for (C) viability as indicated by propidium iodide exclusion. Data is presented as the mean ± SEM for three independent experiments. All groups had viability greater than 98%. *p-value ≤0.05. n.s. means non-significant as determined by one way ANOVA with Tukey’s post-test.
(EPS)

S8 Fig No differences in fungal viability or amount of neutrophil attack in *hog1*∆/∆ or *chs3*∆/∆ mutants. (A-H) *C*. *albicans* strains were treated as described for Fig 6A–6D. (A) Images were analyzed by scoring cells for increased chitin deposition. Data is presented as the percent of total cells with the phenotype for the *cap1*∆/∆ strain. (B) The MFI at equal numbers of sites with and without chitin deposition were obtained for all images from a representative chitin synthase experiment. Results are presented as the average MFI for the pooled sites from that experiment. (C-H) *hog1*∆/∆, *cap1*∆/∆, *chs3*∆/∆ and *chs2*∆/∆ *chs8*∆/∆ chitin synthase strain experiments shown in Fig 6 also had all cells scored for the absence of streptavidin labeling and for viability. Data is presented as the mean ± SEM for three experiments. All groups had viability greater than 99%. *p-value ≤0.05. ***p-value ≤0.001. n.s. means non-significant as determined by one way ANOVA with Tukey’s post-test.
(EPS)

S1 Movie Rapid streptavidin fluorescence loss after neutrophil attachment in live *C*. *albicans*. Streptavidin-Alexa 647-labeled SC5314-GFP was incubated with neutrophils. Timelapses were binned 2x2 and frames were taken at one minute intervals. One representative of 13 timelapses is shown with green, far red and DIC channels.
(AVI)

S2 Movie Rapid streptavidin fluorescence loss after neutrophil attachment in live *C*. *albicans* (far red channel only). Streptavidin-Alexa 647-labeled SC5314-GFP was incubated with neutrophils. Timelapses were binned 2x2 and frames were taken at one minute intervals. One representative of 13 timelapses is shown with the far red channel only.
(AVI)

S3 Movie Rapid Hwp1-GFP fluorescence loss after neutrophil attachment. Streptavidin-Alexa 647-labeled Hwp1-GFP was incubated with neutrophils. Timelapses were binned 2x2 and frames were taken at one minute intervals. One representative of 33 timelapses is shown with the green and DIC channels.
(AVI)

S4 Movie Rapid Hwp1-GFP fluorescence loss after neutrophil attachment (green channel only). Streptavidin-Alexa 647-labeled Hwp1-GFP was incubated with neutrophils. Timelapses were binned 2x2 and frames were taken at one minute intervals. One representative of 33 timelapses is shown with the green channel only.
(AVI)

S5 Movie Rapid extracellular trap deployment by neutrophils. (A-B) WT-FarRed670 was incubated with neutrophils in an imaging dish with CFW and Sytox Green. Timelapses were binned 2x2 and frames were taken at ten minute intervals. The blue, green and DIC channels are shown.
(AVI)

S6 Movie Rapid extracellular trap deployment by neutrophils (blue and green channels). (A-B) WT-FarRed670 was incubated with neutrophils in an imaging dish with CFW and Sytox Green. Timelapses were binned 2x2 and frames were taken at ten minute intervals. The blue and green channels are shown.
(AVI)

S7 Movie Rapid streptavidin fluorescence loss after neutrophil attachment in UV-inactivated *C*. *albicans*. SC5314-GFP was UV inactivated, labeled with streptavidin-Alexa 647 and then incubated with neutrophils. Timelapses were binned 2x2 and frames were taken at one minute intervals. One representative of 27 timelapses is shown with the far red (re-colorized as green) and DIC channels.
(AVI)

S8 Movie Rapid streptavidin fluorescence loss after neutrophil attachment in UV-inactivated *C*. *albicans* (far red channel only). SC5314-GFP was UV inactivated, labeled with streptavidin-Alexa 647 and then incubated with neutrophils. Timelapses were binned 2x2 and frames were taken at one minute intervals. One representative of 27 timelapses is shown with the far red (re-colorized as green) channel only.
(AVI)

S9 Movie Chs3-YFP is recruited to sites of chitin deposition. Chs3-YFP was incubated with neutrophils and Calcofluor white. Timelapses were binned 2x2 and frames were taken at two minute intervals. Representative timelapse of an area with Chs3-YFP recruitment shown. One representative of 11 timelapses is shown with the blue, green and DIC channels.
(AVI)

S10 Movie Sur7-GFP is recruited to sites of chitin deposition. Streptavidin-Alexa 647-labeled Sur7-GFP was incubated with neutrophils and Calcofluor white. Timelapses were binned 2x2 and frames were taken at five minute intervals. One representative of 7 timelapses is shown with the blue, green and DIC channels.
(AVI)

S11 Movie Sur7-GFP is recruited to sites of chitin deposition (green channel only). Streptavidin-Alexa 647-labeled Sur7-GFP was incubated with neutrophils and Calcofluor white. Timelapses were binned 2x2 and frames were taken at five minute intervals. One representative of 7 timelapses is shown with the green channel only.
(AVI)
