# Supplementary material for: Neutrophil Attack Triggers Extracellular Trap-Dependent Candida Cell Wall Remodeling and Altered Immune Recognition
Source: PLoS Pathog. 2016 May 25;12(5):e1005644. doi: 10.1371/journal.ppat.1005644 (PMC4880299; doi:10.1371/journal.ppat.1005644)
Supplement: S2 Fig — BALB/cJ mice were injected in the tail vein with SC5314-GFP and were treated with either PBS or RB6-8C5 antibody via i.p. injection before being sacrificed at day five post infection. (A) Representative images of serial kidney sections from infected mice treated with PBS (A) or RB6-8C5 (B) after being stained with either hematoxylin and eosin (upper images) or Periodic acid schiff (lower images). (C-D) Representative images of kidney homogenates stained with sDectin-1-Fc. Bottom panels show homogenates treated with seconday antibody only. (E) Images were quantified by scoring Candida cell segments for β-glucan exposure (either exposed or non-exposed). Total number of cells found in each category were presented according to mouse treatment group. Three independent experiments were performed. A significant association between PBS treatment and exposed cells and between RB6-8C5 treatment and non-exposed cells was seen, p-value<0.0001 (Fisher’s Exact test). (F-G) Representative images of kidney homogenates stained with anti-1,3-β-glucan antibody. Bottom panels show homogenates treated with secondary antibody only. (H) Images were quantified by scoring as described for (E). A significant association between PBS treatment and exposed cells and between RB6-8C5 treatment and non-exposed cells was seen, p-value<0.0001 (Fisher’s Exact test). Scale bar represents 10 μm. (I-J) Representative images of serial kidney sections from infected mice treated with IgG2a isotype (I) or 1A8 (J) after staining with hematoxylin and eosin or periodic acid-Schiff. The scale bar in the first column of each panel represents 200 μm and in the second column represents 100 μm. (PDF) [file ppat.1005644.s002.pdf]

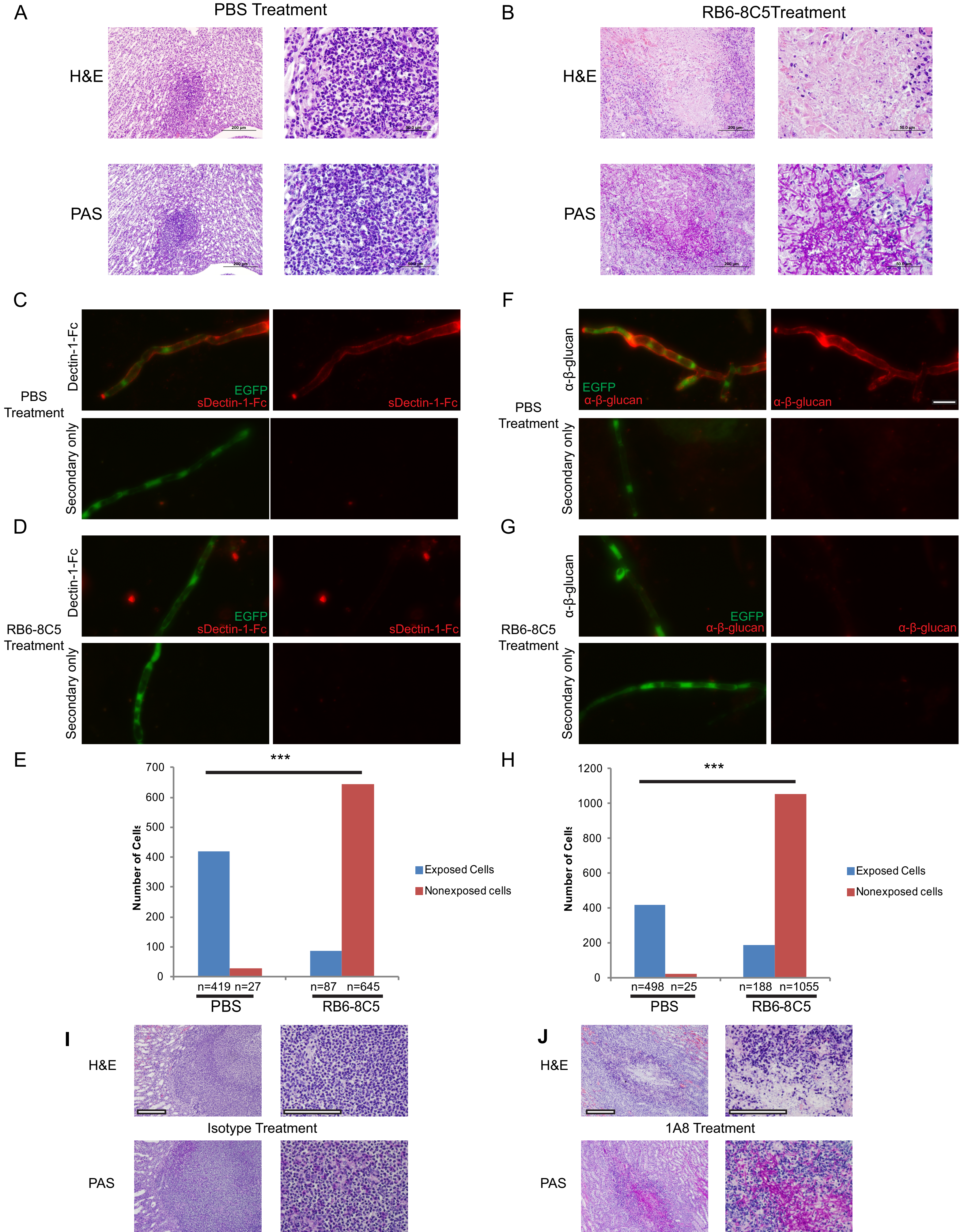
A
PBS Treatment
H&E
PAS
B
RB6-8C5Treatment
H&E
PAS
C
Dectin-1-Fc
PBS
Treatment
EGFP
sDectin-1-Fc
sDectin-1-Fc
Secondary only
D
Dectin-1-Fc
RB6-8C5
Treatment
EGFP
sDectin-1-Fc
sDectin-1-Fc
Secondary only
F
α-β-glucan
PBS
Treatment
EGFP
α-β-glucan
α-β-glucan
Secondary only
G
α-β-glucan
RB6-8C5
Treatment
EGFP
α-β-glucan
α-β-glucan
Secondary only
E
***
Number of Cells
700
600
500
400
300
200
100
0
n=419 n=27
PBS
n=87 n=645
RB6-8C5
Exposed Cells
Nonexposed cells
H
***
Number of Cells
1200
1000
800
600
400
200
0
n=498 n=25
PBS
n=188 n=1055
RB6-8C5
Exposed Cells
Nonexposed cells
I
H&E
Isotype Treatment
PAS
J
H&E
1A8 Treatment
PAS
