# Supplementary figures and images for: Neutrophil Attack Triggers Extracellular Trap-Dependent Candida Cell Wall Remodeling and Altered Immune Recognition
Source: PLoS Pathog. 2016 May 25;12(5):e1005644. doi: 10.1371/journal.ppat.1005644 (PMC4880299; doi:10.1371/journal.ppat.1005644)

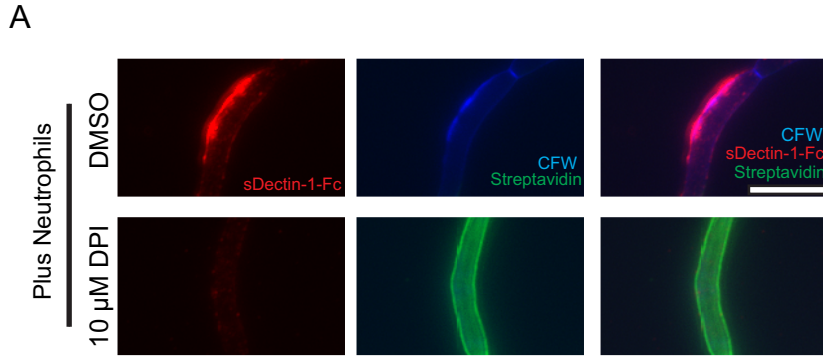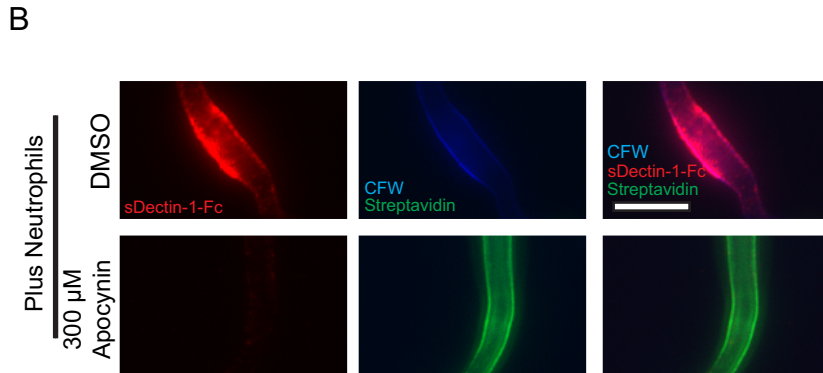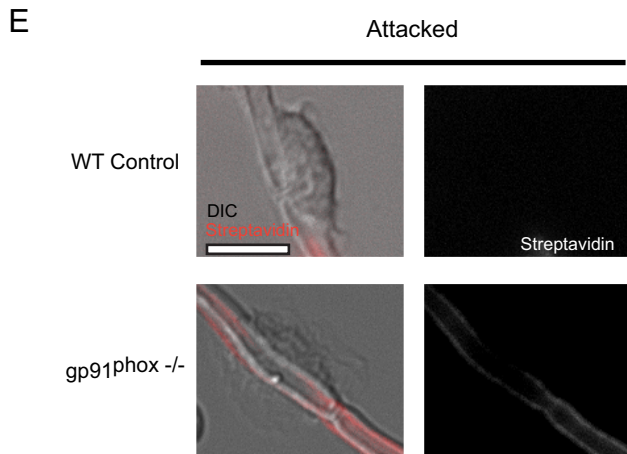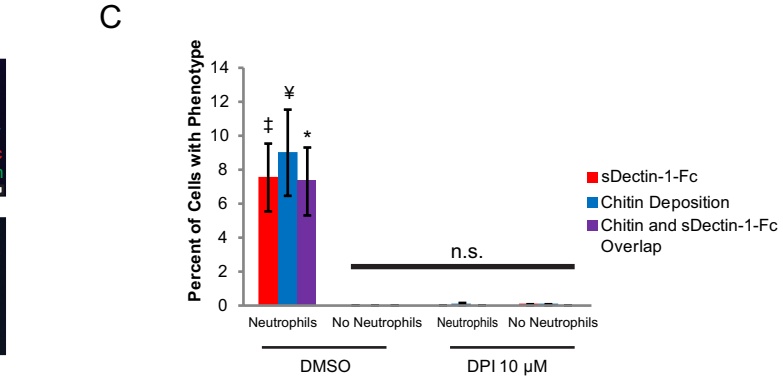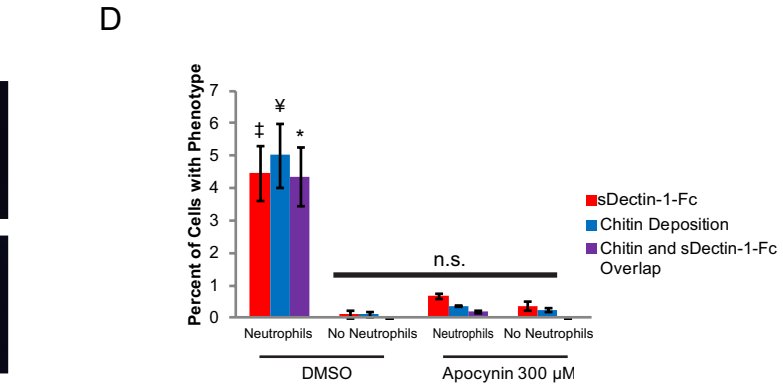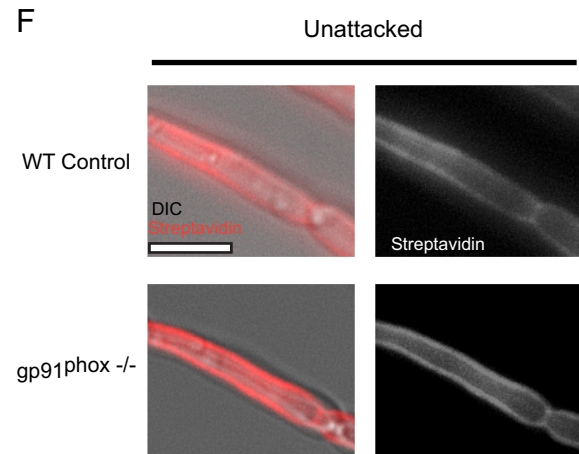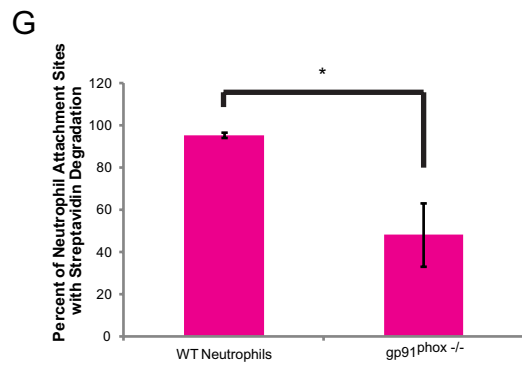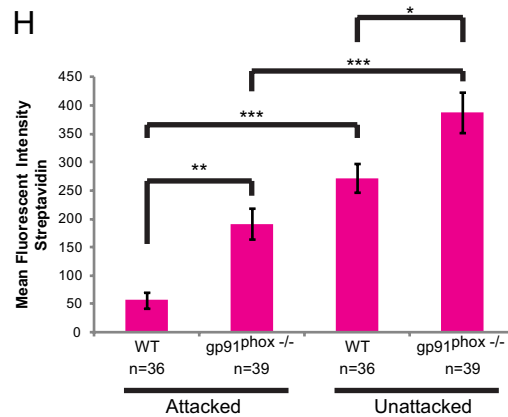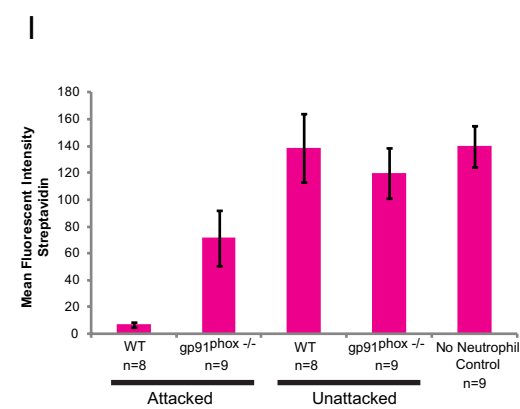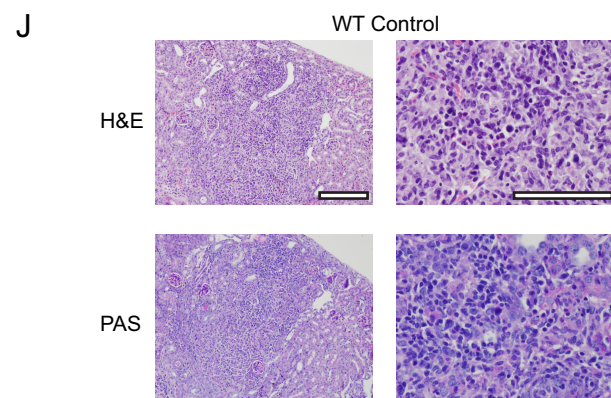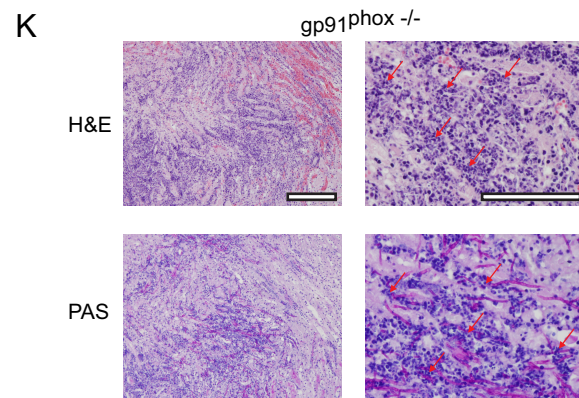

Supplement: S5 Fig — (A-D) Streptavidin-Alexa 647 labeled SC5314-GFP was incubated with neutrophils or alone. Neutrophils were pre-incubated with 10 μM DPI, 300 μM Apocynin or empty vehicle (DMSO) for 10 minutes before being added to fungi. Following incubation, neutrophils were lysed and fungi were stained with sDectin-1-Fc and CFW. (A-B) Representative images are shown. (C-D) Images were analyzed by scoring viable cells for the phenotype indicated and data is presented as the percent of total cells with the mean ± SEM for three independent experiments. ‡ p-value ≤ 0.01 for comparing the sDectin-1-Fc group from DMSO to the 10 μM DPI, 300 μM Apocynin or either no neutrophil groups. ¥ p-value ≤ 0.01 for comparing the chitin deposition group from DMSO to the 10 μM DPI, 300 μM Apocynin or either no neutrophil groups. * p-value ≤ 0.01 for comparing the overlap group from DMSO to the 10 μM DPI, 300 μM Apocynin or either no neutrophil groups. n.s. means non-significant. Comparisons done by one way ANOVA with Tukey’s post-test. (E-I) Streptavidin-Alexa 647 labeled SC5314-GFP was incubated with either C57BL/6J or gp91phox-/- neutrophils in an imaging dish for 30 minutes. (E-F) Representative images for each group. (G) The percent of neutrophil sites with degradation for each group. Data represents the mean ± SEM from three independent experiments, with between 130 and 275 sites scored per experiment. (H) The average MFI in the far red channel at sites of neutrophil attachment. Data represents the mean ± SEM from three pooled experiments where n represents the number of neutrophil attachment sites where MFI was measured. (I) The average MFI for each group, including a no neutrophil control, from a single experiment. (J-K) Representative images of serial kidney sections from either C57BL/6J (J) or gp91phox-/- mice (K) after staining with hematoxylin and eosin or periodic acid-Schiff. The scale bar in the first column of each panel represents 200 μm and in the second column represents 100 μm. (K) [file ppat.1005644.s005.pdf]
